# Assessing alternative crop establishment methods with a sustainability lens in rice production systems of Eastern India

**DOI:** 10.1016/j.jclepro.2019.118835

**Published:** 2020-01-20

**Authors:** Krishna Prasad Devkota, Sudhir-Yadav, C.M. Khanda, Sarah J. Beebout, Bidhan K. Mohapatra, Grant R. Singleton, Ranjitha Puskur

**Affiliations:** a International Rice Research Institute, DAPO Box 7777, Metro Manila, 1301, Philippines; b Present address: Africa Rice Center (AfricaRice), 01 B.P. 2551, Bouaké 01, Cote d’Ivoire; c Orissa University of Agriculture and Technology, Bhubaneswar, India; d Natural Resources Institute, University of Greenwich, Chatham Maritime, Kent, UK

**Keywords:** Sustainability indicators, Dry direct seeded rice, Machine transplanted rice, Environmental footprints, Synergies and trade-off

## Abstract

Sustainability of rice production systems is a prime concern for Asia to maintain food security and to support economic growth. This gain in productivity not only depends on agricultural inputs but also depends on social and environmental factors. To address these emerging issues, new resource- and capital-efficient and profitable technologies have been introduced. The conventional method of rice production (puddling and manual transplanting, PTR) is considered as highly input intensive. As an alternative, dry direct seeded rice (DSR) using seed drill has been promoted to save labor and production costs compared with PTR. Similarly, machine transplanted rice (MTR) has been also considered and promoted in many rice growing countries of South and East Asia. Economic, environmental, and social performances of DSR and MTR (alternative rice establishment technologies) were compared to the PTR using Sustainable Rice Platform (SRP) defined 12 Performance Indicators (PIs) (version 1.0) as a gauge to measure their sustainability. For that, a household survey was conducted on 652 households in Odisha India during 2016. The gaps, i.e., the target to achieve better sustainability, were computed for most of the indicators from the difference between top 10th percentile and the population mean value of the indicator. The results indicated a yield gap of 1.35 t ha^−1^, a profit gap of $273 ha^−1^, labor productivity gap of 21 kg day^−1^, nitrogen (N) use efficiency gap of 22 kg grain kg^−1^ N, phosphorus (P) use efficiency gap of 105 kg grain kg^−1^ P, and water productivity gap of 0.00010 kg grain L^−1^ water in rice production systems in Odisha. Among the compared technologies, MTR results in the highest yield, profit, labor productivity, nitrogen-, phosphorus-use efficiency, and water productivity (at par), and is positive for children’s welfare and the overall energy productivity, indicating better sustainability and has the potential to replace PTR. Direct seeded rice has the highest yield gap (1.57 t ha^−1^; 38%) but has the lowest production cost (can reduce the cost of production by $130 ha^−1^), and the highest greenhouse gas (GHG) reduction potential. SRP PIs are capable for assessing the sustainability of rice establishment technologies except for a few indicators, for example food safety and workers health and safety, which are more applicable to watershed and household level indicators, respectively. The SRP PIs provide scientific evidence and practical impetus for the selection and promotion of sustainable rice production technologies.

## Highlights

• Significant yield, profit, labor productivity, nutrient-use-efficiency gaps exist with current practice of rice production.
• Machine transplanted rice had relatively minimal trade-off than manual transplanting & dry direct seeded rice.
• Optimization of fertilizer, water, pesticide, labor, and production costs are necessary for improving sustainability.
• Direct seeded rice has the highest greenhouse gas reduction potential than conventional and machine transplanted.
• A significant scope exists to further improve the sustainability indicators to well define sustainability of rice production.

## Graphical abstract

**Figure:**
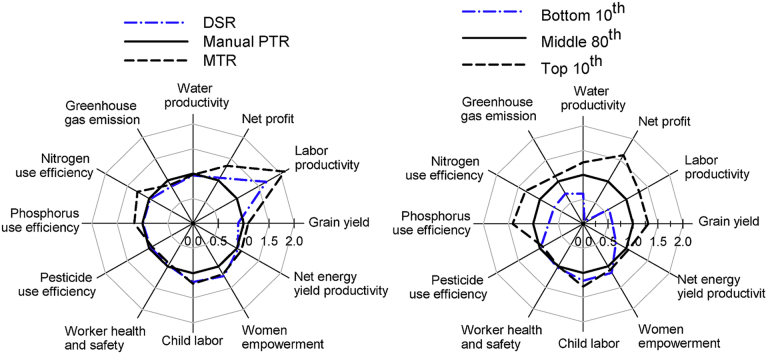

## 1 Introduction

As a staple food, rice plays a central role in food security and economic growth of India. It is grown in more than one-fifth of the total gross cropped area and contributes more than one-fourth of the total calorie intake (Mohanty and Yamano, 2017). In the last five decades, India has tripled its rice production through the use of high-yielding varieties, improved management practices and agricultural support programs for the farmers, and is now a rice-surplus country. However, seven states namely Odisha, West Bengal, Bihar, Eastern Uttar Pradesh, Assam, Jharkhand, and Chhattisgarh account for more than 55% of the total rice area while contributing less than 50% to total production (Mohanty and Yamano, 2017) and has experienced a stagnated productivity growth for the past two decades. In Odisha state, rice is synonymous with food, and is grown in 4.4 million ha accounting for 91% of the area under cereals and contributes about 94% of total cereal production in the state (Das, 2012). The state’s food security, economy, nutrition, and poverty alleviation are directly linked with rice.

Sustainability is “meeting society’s present needs without compromising the ability of future generations to meet their own needs” (Ghelichkhan et al., 2018). Maintenance of environmental quality with economic advantages over humankind’s place are the roots of sustainability (Bell and Morse, 2012). Inclusion of social dimension is important to ensure balancing benefits and sustainability (Musumba et al., 2017). Several methods to select indicators and pillars for measuring sustainability has been developed (Marinus et al., 2016), while the three pillars, i.e., economic viability, environmental responsibility, and social acceptability” have increased public awareness and investigations of sustainability of alternative technologies (Ghelichkhan et al., 2018; Marinus et al., 2016). In India, manual transplanting in puddled soil (PTR) is the most common method for rice establishment and covers about 77% of the rice area (Rao et al., 2007). Availability of water for irrigation and cheap labor had historically encouraged farmers to use this practice. These resources, however, are becoming increasingly scarce and expensive, therefore threatening the sustainability and the viability of PTR system. In recent years, much research has highlighted the challenges with PTR including high yield gap (Lobell et al., 2009), low water and nutrient productivity (Humphreys et al., 2010; Sudhir-Yadav et al., 2011b), high greenhouse gas (GHG) emission (Wassmann et al., 2004), low energy use efficiency (Quilty et al., 2014), rising labor scarcity (Devkota et al., 2019b, Devkota et al., 2019a), and associated increased wages (Sudhir-Yadav et al., 2017; Bandumula et al., 2018). Together, these changes have increased the workload of women (Akter et al., 2017), and led to higher cost of cultivation and reduced profitability (Ditzler et al., 2018).

A sustainable intensification and rice production growth rate are the prime concerns for the country. To maintain food security and support economic growth; gains in productivity have to essentially come with efficient use of all resources, including land, labor, water, energy, and chemicals, along with a lower environmental footprint and building resilience (Rockström et al., 2017). These emerging issues warrant a paradigm shift in the traditional rice-growing system being practiced in eastern India, giving way to more resource- and capital-efficient and profitable systems that we need to feed future generations. Dry direct seeded rice (DSR; dry direct seeding of seed using seed-cum-fertilizer drill after dry tillage of the soil) and machine transplanted rice (MTR; 18–22 day old seedling transplanting in puddled soil using transplanting machine) have extensively been evaluated and promoted in Odisha and other parts of India and Southeast Asia as alternative sustainable intensification technologies to address the rising scarcity of labor, reducing production costs and the issue of depletion of natural resources especially water (CSISA, 2017), thus, as a means to balance the environmenal, economic, and social indicators. Direct seeded rice can improve land productivity, reduce costs, enhance household income, increase yield, and reduce fertilizer and land preparation costs (Mishra et al., 2017). Compared to PTR, DSR saves up to 30% of the total production cost (Alam et al., 2018), 30–50% irrigation water (Sudhir-Yadav et al., 2011a,b), and promotes mechanization which reduces women drudgery, labor, production costs, and energy (saves 1500 MJ ha^−1^ season^−1^) (Gathala et al., 2014; Kumar and Ladha, 2011)Laik et al., 2014. Further, DSR allows for timely crop establishment (Kumar and Ladha, 2011), and reduces tillage which prevents the destruction of soil structure caused by puddling, and decreases the dependency on labor (Timsina and Connor, 2001). For these reasons, farmers are gradually shifting rice cultivation from PTR to DSR system in Eastern Indo-Gangetic Plains (IGP) (CSISA, 2017). A similar change to wet method of direct seeded rice (wet-DSR) has been reported in China (Wang et al., 2017). In the IGP, the India government supports for MTR and DSR through subsidizing the cost of transplanting and seed-drill machines (CSISA, 2017).

To our knowledge, there have been no attempts to use a common holistic framework to assess the economic, environmental and social indicators (sustainability) of these alternate crop establishment technologies being promoted as the drivers for sustainable intensification of rice in India and elsewhere. The Sustainable Rice Platform (SRP; http://www.sustainablerice.org), is such a holistic multi-stakeholder platform co-convened by United Nations Environment (UN-Environment) and the International Rice Research Institute (IRRI). Its aim is to promote sustainability in rice production and profitability with reduced environmental footprint and with better social values, and sustainability in rice consumption, supply and trade in global rice sector (SRP, 2015a, 2015b). It has defined 12 Performance Indicators (PIs) to assess the sustainability of rice production based on economic, environmental and social indicators as presented in Supplementary Information (SI) Table 1.

**Table 1:** Characterization of production inputs for three different rice establishment methods in Odisha, India, 2016.

Production inputs*	DSR	Manual PTR	MTR
No. of households surveyed	250	260	142
Total land holding (ha household^−1^)	1.41 ± 1.07	1.10 ± 0.73	1.61 ± 0.98
Rice area for comparison (ha household^−1^)	0.54 ± 0.34	0.45 ± 0.24	0.61 ± 0.41
Nursery establishment date	–	23 June ± 12	28 June ± 13
Sowing (DSR)/transplanting date	27 June ± 16	15 July ± 12	20 July ± 14
Harvesting date	4 Dec ± 16	9 Dec ± 9	10 Dec ± 11
Seedling age (day)	–	23 ± 5	22 ± 5
Maturity duration (day)	160 ± 22	170 ± 14	165 ± 15
Most common varieties planted (figure in parenthesis indicated percent farmers growing respective variety)	Pooja (39), Rani (29), Swarna Sub-1 (19), Swarna Masuri (8), RGL (2), Barsha (1)	Pooja (39), Swarna Masuri (30), Rani (12), Swarna Sub-1 (5), Sarala (3), Barsha (2), 1014 (2), 1018 (2), Jagabalia (2), Moti (1)	Swarna Masuri (31), Pooja (25), Rani (25), Swarna Sub-1 (14), Praktikhya (2), Lalat (1)
Seed rate (kg ha^−1^)	41 ± 12	54 ± 10	51 ± 9
Elemental N (kg ha^−1^)	77 ± 29	88 ± 38	75 ± 29
Elemental P (kg ha^−1^)	19 ± 9	21 ± 8	20 ± 8
Elemental K (kg ha^−1^)	39 ± 22	46 ± 22	44 ± 22
No. of labor day ha^−1^	74 ± 32	125 + 42	73 ± 36
Land preparation	Non-puddled	Puddled	Puddled
Irrigation applied (mm)	187 ± 192	310 ± 238	386 ± 221
Rainfall (mm)	918 ± 378	796 ± 278	821 ± 242
No. of irrigation application	2.4 ± 2.4	3.8 ± 3.0	4.8 ± 2.8
No. of fertilizers basal application	1.7 ± 0.6	1.7 ± 0.6	2.0 ± 0.7
No. of topdressing	3.0 ± 1.1	3.0 ± 1.1	3.0 ± 1.1
No. of pesticide application	2.0 ± 1.2	1.5 ± 1.4	2.5 ± 1.3
Total variable cost of production ($ ha^−1^)	502 ± 120	632 ± 132	602 ± 127

*Values are mean ± Standard deviation (SD). DSR = dry seeded, PTR = manual puddled transplanted, MTR = machine transplanted rice.

Intensification of rice production has helped to increase food security but there is a widely recognized need to assess the sustainability of such production systems. A previous study on quantification and comparison of economic and environmental indicators of SRP indicators in lowland rice production in six Asian countries indicates that significant improvement in the economic and environmental indicators is possible by promotion and widescale adoption of sustainable practices in the rice value chain and input (fertilizer, water, pesticide, seed, labor) optimization (Devkota et al., 2019b, Devkota et al., 2019a). Similarly, “three controls technology” (3CT; the efficient use of nutrients especially N; reduced unproductive tillers and lodging; and reduced spray of fungicide and insecticide; Zhong et al., 2010), one approach in China for sustainable rice production has proved to be effective in Guangdong Province for decreasing N fertilizer rate (Wang et al., 2017). The potential to reduce water and pesticide use, optimization of crop production inputs N-, P-, and K- fertilizers, seed rate, and pesticide application time and number, the option for increasing mechanization for reduced labor and child, and women empowerment for sustainably closing yield and profit gaps with reduced environmental footprint, which ultimately determines the sustainability of rice production system, could differ with establishment methods (DSR, manual PTR and MTR). However, assessment of different rice establishment methods with sustainability indicators considering yield, labor use, pesticide and fertilizer application, machinery use, energy consumption and profitability also considering child and women are lacking. Thus, the objectives of the current study are to understand trade-offs among different economic, environmental and social indicators of sustainability with alternate establishment methods, and to verify the applicability of SRP defined PIs for gauging sustainability of rice establishment methods.

## 2 Materials and methods

### 2.1 Household survey

**Fig. 1:**
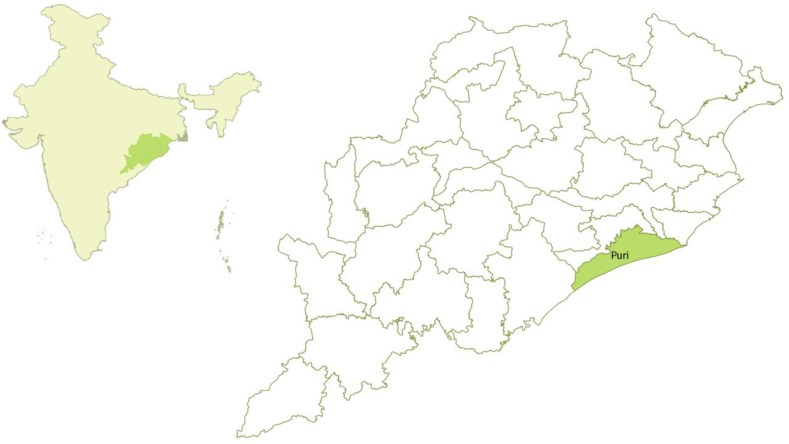
Map of the study site (Puri district of Odisha, India).

For the comparison of economic, environmental and social indicators across three rice establishment methods, paper-based questionnaire (SI-Appendix 1) for the computation of 12 SRP defined PIs (version 1.0) and energy-use efficiency (proposed as a potential13^th^ indicator) was used. The survey questionnaires were derived from the set of SRP indicators (SRP, 2015a) which went through open public review for content reliability and received comments from more than 90 public and private agencies and stakeholders involved in the rice research and development across the world. Before starting the survey, the questionnaire was pre-tested and validated for the local context by establishing a panel of experts. A household survey was conducted during January–February 2016 in Puri district of Odisha (Fig. 1). This district was purposively selected as it represents most intensive rice growing coastal belt regions of the state with a high rate of adoption of new technologies. Stratified random sampling was used to select the blocks (8) and villages (30) within Puri district. For household selection and sampling from blocks and villages, the database of DSR and MTR adopter farmers in each block and villages were collected from the State Agricultural Department and IRRI’s ongoing projects in the region. From the database, for all three establishment methods, households which represents most of the rice growing households of the state considering total land holding of not more than 6 ha and rice area of not more than 3 ha, representative farmers in terms of input use and crop management practices were selected purposively for the survey. Additionally, as DSR and MTR are still comparatively new technologies, farmers adopting these technologies continuously for more than 2 years were chosen proportionately from all blocks and villages. The total sample size for survey was 652 households with 250 DSR, 260 PTR and 142 MTR in Puri district of Odisha.

### 2.2 Data collection, coding, and cleaning

The data were collected using a semi-structured questionnaire (SI-Appendix 1) designed for the computation of 12 PIs as defined by the SRP PIs (version 1.0). Additionally, basic demographic and farm characterization information was collected during the interview. Farmers were asked to recall all information of the most recent previous “kharif” (wet) season of 2015 required for the calculation of each indicator. The approximate depth of irrigation water in each irrigation (cm) and the number of irrigations applied were asked, from which water productivity was calculated. Farmers also were asked for the amount and timing of all input materials used, e.g., seed, fertilizer, herbicide, insecticide, fungicide, rodenticide, and molluscicides. Women in the households answered the questions in the section on women’s decision making. During data cleaning, missing data regarding the cost of specific field operations for rice production (e.g., field preparation, seeding or transplanting, nursery cost for the PTR and MTR, harvesting, threshing, and cleaning) were set at their respective practices average cost ha^−1^ from the other farmers in their block. Grain price was asked during the survey and the nominal straw price considered, i.e., 1% of the grain price as many of the farmer burn the straw in this district. Straw yield was computed using a 40% harvest index of rice (Kar et al., 2018; Li et al., 2012).

### 2.3 Calculation of SRP performance indicators (PIs)

All 12 performance indicators, i.e. net profit, labor productivity, grain yield, food safety, water productivity, nitrogen-use efficiency (NUE), phosphorus-use efficiency (PUE), pesticide-use efficiency, GHG emission, health and safety, child labor, and women empowerment were calculated as defined by SRP (SRP, 2015a). A detailed explanation of computation of all SRP PIs is given in Appendix 2 (SI). Though SRP used the term “water use efficiency” they mean it as “water productivity”. To keep consistency, we have used the term water productivity in this paper. Water productivity (kg paddy L^−1^ irrigation + rainfall) was computed by dividing the amount of grain yield by total amount of irrigation and rainfall water (L). For each farmer,the total amount of irrigation water applied and the amoount of rainfall received during the crop growing period was computed. The amount of irrigaiton water computed from the number of irrigation and the approximate depth of irrigation applied (Devkota et al., 2019b, Devkota et al., 2019a) from the start of field preparation to crop harvest, and the total amount of rainfall computed from crop sowing (for DSR) and transplanting (for manual PTR and MTR) to harvest (crop growing period) using the daily rainfall data obtained from the Odisha Meteorological Stations. The pesticide use efficiency was calculated using the SRP scorecard, which is based on the number and the timing of application of different categories of pesticide, i.e., herbicide, insecticide, fungicide, rodenticide, molluscicide, and avicides; the application of personal protective measures; and the follow-up of other safety rules and regulations (SRP, 2015a; Stuart et al., 2018). Bird control was also included in the computation of the pesticide use score. For the sustainability assessment, the scorecard values were categorized (out of a possible score of 100) as: “Gold” if the scorecard value  ≥ 80, “Acceptable” if 65–80, “Tolerable” if 50–65, and “Unsustainable” if  ≤ 50. Greenhouse gas emission was computed using the formulas given by IPCC (Dong et al., 2006) and Stuart et al. (2018). The indicator of food safety requires laboratory analysis of heavy metals, e.g., arsenic, cadmium, mercury, chromium, and lead, and pesticide residues and we did not have access to a high-quality service laboratory for these analyses, it was computed based on the farmer’s response to the reported problem in the rice field (SRP, 2015a). Three social indicators, i.e., worker health and safety, child labor and women empowerment were computed using the SRP scorecard (SRP, 2015a) and for the assessment of sustainability, the scorecard values were ranked (out of a possible score of 100) as, “Good” if the scorecard value  ≥ 80, “Fair” if 50–80, “Poor” if  ≤ 50.

### 2.4 Energy use efficiency

Besides the computation of the 12 performance indicators, we also computed and compared the energy use efficiency (a potential 13th indicator for consideration by SRP) among three rice establishment methods. For all three establishment methods, four energy use efficiency indicators, i.e., agronomic energy input (AEI), energy output/grain yield energy (GYE), net energy balance (NEY), and net energy yield productivity (NEYp) were computed (SI-Table 2 and SI-Appendix 2). Questions on the type of machines used, technical specification of machineries including motor capacity (horsepower), type of fossil fuel consumption during operation, and the total time (h) taken for the particular machinery for particular operations, e.g., laser land leveling, nursery bed preparation, main field preparation, and direct seeding or transplanting, harvesting (manual or reaper or combine), and threshing in the surveyed rice area were asked (SI-Appendix 1). When computing AEI, energy consumed by machines during these field operations were computed using the modifying formula provided by Eskandari and Attar (2015) as these services were mostly provided by the service providers and thus the efficient life-time of the machine and the power-to-weight ratio (some machines are old) are beyond their control and can vary from one service provider to another. Also for the same land area, planting and harvesting method (manual or machinery), crop yield per unit area, number and the approximate depth of irrigation (volume of irrigation water in m^3^ ha^−1^ during the crop growing period computed), number and type (male or female) of manual labor (person-hour) starting from nursery and land preparation to harvesting and threshing, the amount of seed, amount and type (nitrogen, phosphorus, potassium) of fertilizers, and amount and type of pesticides (herbicide, insecticide, fungicide, rodenticide, molluscicide) used, and amount of compost applied were recorded through recall during survey and computed (SI-Appendix 2).

### 2.5 Data analysis

All indicators were computed using the Excel Version of the Field Calculator. The attainable (exploitable) yield gap was computed from the difference between the mean of top 10th percentile and the population mean, and the percent yield gap was calculated by dividing this difference by the mean value of the top 10th percentile. Using the same approach, attainable gaps in other indicators, i.e., profitability, labor productivity, nitrogen- and phosphorus-use efficiency, water productivity, and greenhouse gas emission also were computed and displayed in frequency distribution indicating top and bottom percentile values. To compare the sustainability and trade-offs among performance indicators (including energy) and input use among three establishment methods and three yield gap category farmers (top 10th, middle 80th and bottom 10th), radar/spider diagrams (with the values on each spoke normalized to visually comparable units) were used. Descriptive statistics (mean and standard deviation) are presented wherever it is applicable. For the comparison of establishment methods across the PIs, analysis of variance (ANOVA) was computed considering the number of farmers as replication and establishment method as treatment using the unbalanced generalized linear model (GLM) function of R version 3.5.

## 3 Results

### 3.1 Production inputs among three rice establishment methods

The application of the production inputs (NPK fertilizers, seed and the labor) varied and was highest in manual PTR (Table 1). Farmers used similar varieties for PTR, DSR and MTR. However, the number of varieties grown under new establishment methods (DSR and MTR) were less (7) than under PTR (11).

Interestingly, the seed rate with DSR (41 kg ha^−1^) was significantly lower than MTR (51 kg ha^−1^) and PTR (54 kg ha^−1^). Seeding window of DSR was similar to nursery establishment for MTR or PTR. Due to early seeding, the water input as rainfall was maximal in DSR followed by MTR and PTR. The N fertilizer rate used in DSR and MTR were lower than in PTR, and in overall the N rate applied in all establishment methods was lower than the recommended N rate for rice in Odisha. Farmers apply only 50–60% of the recommended rate of fertilizer in irrigated rice and 25–30% of the recommended fertilizer rate in favorable rainfed rice in Puri district. Around 97% of farmers applied all N, P and K fertilizers, 29% of farmers applied urea fertilizer also as basal, 47% of farmers applied muriate of potash fertilizer at topdressing, and 4% of farmers applied diammonium phosphate as well in topdressing. The farmers generally applied 1.8 ± 0.6 fertilizer types at basal, and 3.0 ± 1.1 types of fertilizers at topdressing. Most of the farmers (99%) applied fertilizers in splits (62% one split and 37% in two split applications at top-dressing). The MTR farmers applied the highest number and amount of irrigations followed by PTR. The lowest water use was by the DSR farmers. On average, there was more than one pesticide application for all establishment methods and the highest number of applications in MTR.

### 3.2 Grain yield and yield gap

**Fig. 2:**
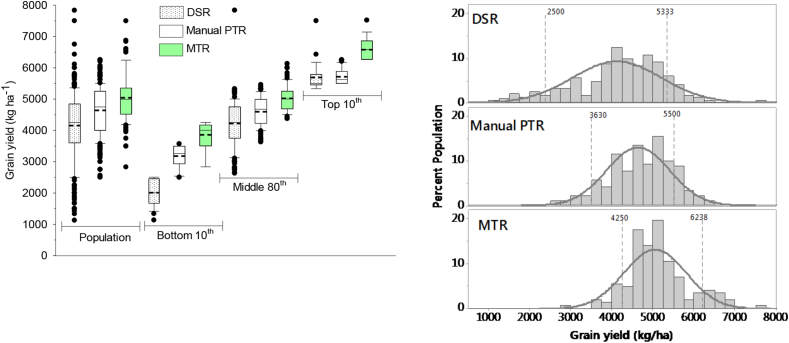
Left side: Grain yield of the mean of three different establishment methods (population), and bottom 10th, middle 80th, and top 10th percentile of farmers in Odisha, India, 2016. The short-dotted line inside the box is the mean and the black solid straight line the median. N = 652, where DSR = 250; Manual PTR = 260; and MTR = 142, DSR = dry seeded rice, PTR = puddled transplanted rice and MTR = machine transplanted rice. Right side: The frequency distribution and normality curve and two vertical dotted lines indicating the 10th and 90th percentile and their values of each establishment method.

The rice yield was highest under MTR (5.05 ± 0.75 t ha^−1^) followed by PTR (4.64 ± 0.77 t ha^−1^) and the lowest in DSR (4.12 ± 1.01 t ha^−1^). The overall mean yield gap of rice in Odisha was 1.35 t ha^−1^. Among different technologies, the highest yield gap of 1.6 t ha^−1^ (38%) was in DSR, followed by MTR at 1.5 t ha^−1^ (30%), and the lowest at 1.1 t ha^−1^ (23%) for manual PTR (Fig. 2). As overall comparison, a significant number of farmers (21%) achieved the yield in lower range with DSR and higher range with MTR (23%); indicating a risk of yield penalty with DSR and scope for further increasing yield with the adoption of MTR.

### 3.3 Cost of production, net profit, and labor productivity

**Fig. 3:**
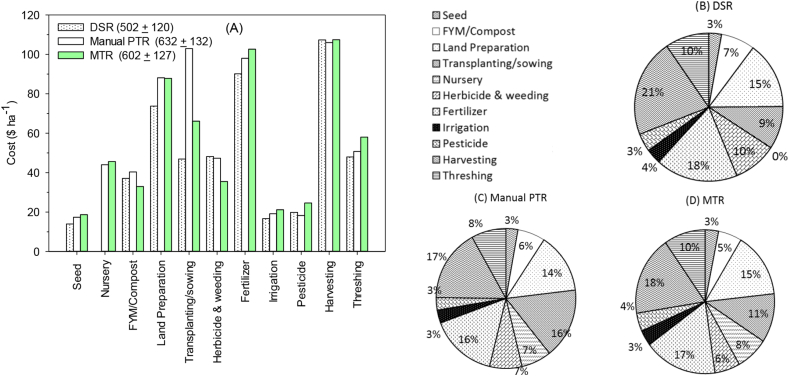
Cost of rice production operations ($ ha^−1^) for three establishment methods (A) and the percentage cost for each operation for dry seeded rice (DSR, B), manual puddled transplanted rice (PTR, C) and machine transplanted rice (MTR, D) in Odisha, India, 2016. Values in parenthesis in Fig. A, total production costs ± SD ($ ha^−1^).

Dry direct seeded rice ($502 + 120 ha^−1^) had the lowest production cost; $100 ha^−1^ (17%) lower than MTR ($602 + 127 ha^−1^) and $130 ha^−1^ (21%) lower than PTR ($632 + 132 ha^−1^) (Fig. 3). In terms of costs per kg grain production, MTR was lowest of $0.12 kg^−1^ grain followed by DSR ($0.13 kg^−1^ grain), and PTR ($0.14 kg^−1^ grain). Most of the cost saving in DSR came from avoiding nursery establishment ($44 ha^−1^) and reduced labor in crop establishment ($56 ha^−1^). Crop establishment (nursery + transplanting/sowing costs) under DSR required only 9% of the total cost, while it required 19% with MTR and 23% in manual PTR (Fig. 3).

**Fig. 4:**
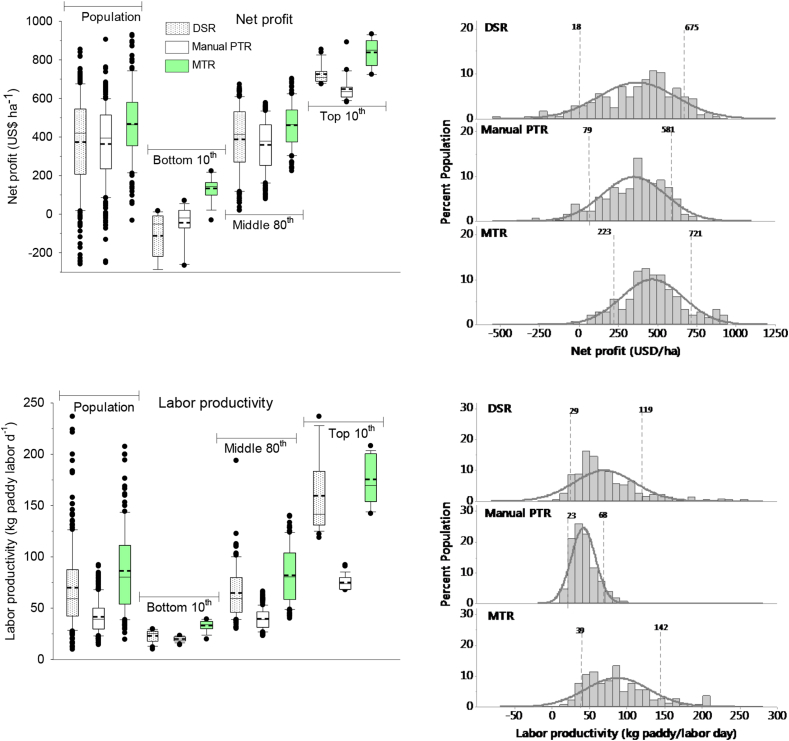
Left side: Net profit (A) and labor productivity (B) among the bottom 10th, middle 80th, top 10th percentile of farmers in dry seeded (DSR), manual puddled transplanted (PTR) and machine transplanted rice (MTR) in Odisha, India, 2016. The short-dotted line inside the box is the mean and the black solid straight line the median values. Right side: The frequency distribution and normality curve and two vertical dotted lines indicating the 10th and 90th percentile and their values of each establishment method.

In Odisha, the highest net profit, in top decile farmer ($761ha^−1^), and labor productivity (108 kg grain day^−1^) were with MTR followed by DSR (662 and 86, respectively) and PTR (568 and 55, respectively) (Fig. 4). With MTR, the average net profit ($468 ha^−1^), was higher by 34% and 26% than PTR ($350 ha^−1^) and DSR ($371 ha^−1^), respectively. The coefficient of variation of net profit was the highest with DSR (67%) followed by PTR (57%) and the MTR (42%). Compared to PTR, MTR and DSR both decreased the number of labor use by 42%, and increased the labor productivity by 45 and 28 kg grain day^1^, respectively (Fig. 4). The frequency distribution for both net profit and labor productivity showed a greater number of farmers in top 10th percentile in MTR, while less (for net profit) and no farmers (in labor productivity) in top 10th percentile in manual PTR (Fig. 4).

### 3.4 Nitrogen-, phosphorus-use efficiency, water productivity and greenhouse gas emission

**Fig. 5:**
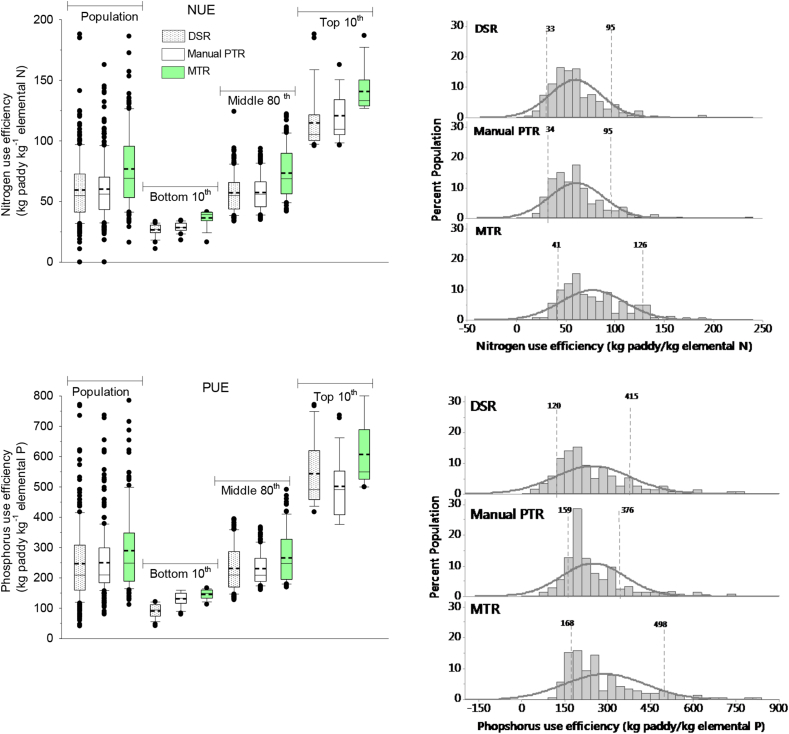
Left side: Nitrogen- and phosphorus-use efficiencies among the bottom 10th, middle 80th, top 10^th^percentile of farmers in dry seeded (DSR), manual puddled transplanted (PTR) and machine transplanted rice (MTR) farmers in Odisha, India, 2016. The short-dotted line inside the box is the mean and the black solid straight line the median. Right side: The frequency distribution and normality curve and two vertical dotted lines indicating the 10th and 90th percentile and their values of each establishment method.

**Fig. 6:**
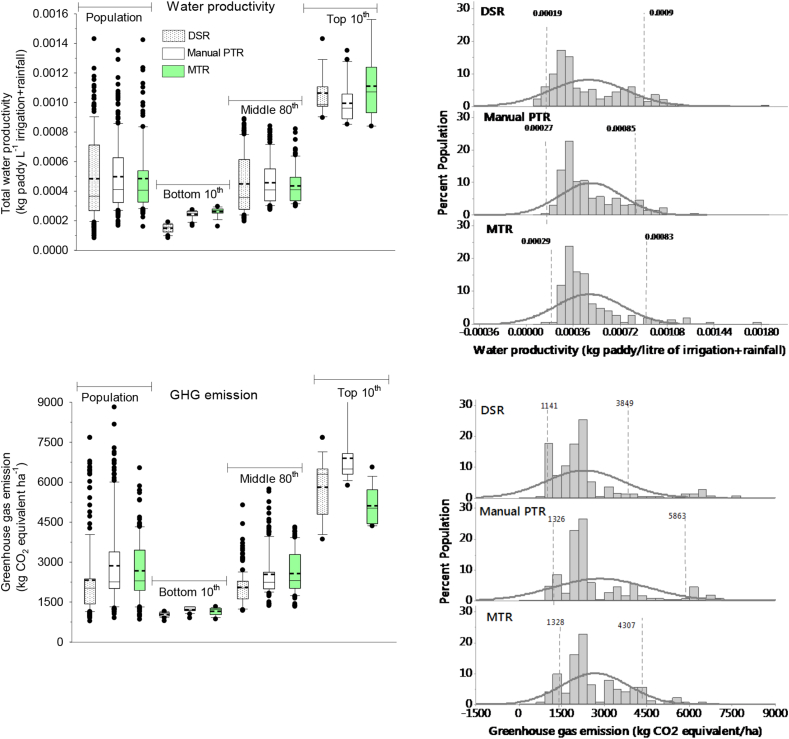
Left side: Water productivity and greenhouse gas (GHG) emission among the bottom 10th, middle 80th, and top 10th percentile of farmers in dry seeded (DSR), manual puddled transplanted (PTR) and machine transplanted rice (MTR) in Odisha, India, 2016. The short-dotted line inside the box is the mean and the black solid straight line the median. Right side: The frequency distribution and normality curve and two vertical dotted lines indicating the 10th and 90th percentile and their values of each establishment method.

Both N- and P-use efficiencies were highest in MTR followed by at par in DSR and PTR (Fig. 5). Water productivity was similar among the three establishment methods (Fig. 6), however, the amount of water input per kg grain was highest for DSR (2980 kg kg^−1^ grain) and similar for MTR and PTR (2400  kg kg^−1^ grain). The highest GHG emission was from the PTR (17% higher than DSR) followed by MTR (5% higher than DSR) (Fig. 6). The frequency distribution for both N- and P-use efficiencies and water productivity showed a greater number of farmers towards top 10th in MTR (Fig. 5), while for GHG emission, manual PTR has a greater number of farmers towards top 10th percentile (Fig. 6).

### 3.5 Pesticide use efficiency

Based on the SRP PIs, the majority of the farmers fall under the category of gold and acceptable pesticide applicator in Odisha (Table 2). A higher proportion of PTR farmers were under the gold category followed by DSR and MTR.

**Table 2:** Number of farmers in different category on pesticide application (based on SRP scorecard method) and the percentage (figure in parenthesis) of farmers using pesticides to control weeds, insects, diseases, rats, and mollusks in Odisha, India, 2016.

Establishment method	Gold	Acceptable	Tolerable	Unsustainable
DSR	87 (35)	130 (52)	29 (15)	4 (2)
Manual PTR	112 (43)	119 (46)	22 (8)	7 (3)
MTR	43 (30)	66 (46)	26 (18)	7 (5)
Grand total	242 (37)	315 (48)	77 (12)	18 (3)

Overall, 21% of farmers did not apply any pesticide, 16% of farmers applied pesticide one time, 25% of farmers applied pesticides twice, 30% applied them three times, and 9% applied them more than three times. When farmers applied pesticides, 67% of farmers applied herbicides, 69% insecticides, 42% of farmers applied fungicides, and 4% of farmers applied rodenticides (SI Table 3). Less than 1% of farmer applied molluscicide, and 4% of farmer (5, 2 and 7% respectively of DSR, PTR, and MTR, respectively) reported birds as a problem, and they did not apply any baiting except the manual method of bird scaring.

**Table 3:** Number and percentage (in parenthesis) of farmer’s in three rice establishment methods in different category in health and safety, child labor, and women empowerment) (based on SRP scorecard) in Odisha, India, 2016.

Establishment method	Good	Fair	Poor

**Worker health and safety**			
DSR	1 (0)	114 (46)	135 (54)
Manual PTR	1 (0)	153 (59)	106 (41)
MTR	2 (1)	55 (39)	85 (60)
**Overall**	**4 (1)**	**322 (49)**	**326 (50)**
**Child labor**			
DSR	97 (39)	13 (5)	140 (56)
Manual PTR	70 (27)	10 (4)	180 (69)
MTR	64 (45)	4 (3)	74 (52)
**Overall**	**231 (35)**	**27 (4)**	**326 (60)**
**Women empowerment**			
DSR	26 (10)	33 (13)	191 (76)
Manual PTR	17 (7)	27 (10)	216 (83)
MTR	9 (6)	21 (15)	112 (79)
**Overall**	**52 (8)**	**81 (12)**	**519 (80)**

Note: In SRP scorecard, sore of >80 = Good; 50–80 = Fair; and <50 = poor for all three indicators.

### 3.6 Food safety and worker health and safety

#### 3.6.1 Food safety

From the computation of food safety indicator as defined by SRP, only 3% of the farmers responded that they are aware of risks in food safety from cadmium, chromium, mercury, and lead, and none of them could specify the name of the heavy metal which is problematic in their location. This response was similar for crop establishment methods (varied between 2 and 5%).

#### 3.6.2 Worker health and safety

Fifty percent of the farmers practice poor to fair health safety mostly due to the poor management of pesticides (Table 3). Only 40% of the pesticide applicators had received training in the last 5 years. Lack of knowledge or not following the safety rules, such as washing and changing clothing and equipment followed by the incidence of a work-related accident, applicators restriction, calibration of equipment, training for the pesticide applicators and re-entry time into the crop after spraying (SI Table 4) are the major reasons for the low score (poor category) of this indicator. Hardly any pesticide applicators used a complete set of personal protective equipment whilst spraying. Interestingly, 70% of the farmers did not report work-related health incidences, but the same proportion of the farmers did not have first aid kits (SI Table 5). Regarding the seed drill, more than 80% of farmers responded that they outsourced the service (from service providers from the same or nearby village).

**Table 4:** Farmers’ scores (based on the SRP scorecard) on variables for defining women empowerment for rice production under different establishment methods in Odisha, India, 2016.

SN	Variables for defining women empowerment*	DSR	Manual transplanting (PTR)	MTR	Overall	p-value
1	Women’s control over decisions regarding household agricultural production	3.22 ± 4.00a	2.20 ± 3.45b	2.55 ± 3.57 ab	2.67 ± 3.72	0.00
2	Women’s role and control over the decision regarding selected technology	3.76 ± 2.16a	2.48 ± 2.50b	2.75 ± 2.50b	3.03 ± 2.45	0.000
3	Change for women with new technology	6.68 ± 4.72b	5.96 ± 4.92b	7.89 ± 4.10a	6.66 ± 4.72	0.000
4	Women’s satisfaction regarding new technology	3.82 ± 2.13a	2.38 ± 2.50b	3.35 ± 2.36a	3.14 ± 2.42	0.000
5	Women’s satisfaction regarding their labor input	6.73 ± 4.26a	4.87 ± 4.36b	6.87 ± 4.09a	6.02 ± 4.36	0.000
6	Women’s access to information and capacity building for new technology	2.63 ± 3.79a	1.87 ± 3.46b	2.17 ± 3.05 ab	2.23 ± 3.52	0.049
7	Women’s access to seasonal resources for farm activities	3.44 ± 4.10a	2.58 ± 3.84b	3.27 ± 3.58 ab	3.06 ± 3.90	0.034
8	Women’s control over decision-making regarding household income	3.62 ± 4.32 ab	2.77 ± 4.03b	3.87 ± 4.06a	3.34 ± 4.17	0.015
9	Women’s control over their personal income	2.14 ± 3.45	1.92 ± 3.49	2.76 ± 4.01	2.19 ± 3.60	0.081
10	Women’s participation in collective decision-making	2.26 ± 3.01	1.97 ± 3.00	1.92 ± 2.64	2.07 ± 2.93	0.407
11	Violence against women	9.56 ± 2.06	9.35 ± 2.48	9.44 ± 2.31	9.45 ± 2.29	0.572

*Values are mean ± SD. Each question had the maximum score of 10. DSR = dry seeded, PTR = manual puddled transplanted, MTR = machine transplanted rice. Similar letter in the table indicate non-significant difference at p < 0.05 in each row.

**Table 5:** Mean energy input in various inputs and operations, and energy use efficiencies (agronomic energy input (AEI), net energy (NEY), and energy productivity (NEYp)) for three rice establishment methods for the surveyed household in Odisha, India, 2016.

SN	Operation (GJ ha^−1^)*	DSR	Manual PTR	MTR	p-value
1	Laser land leveling	1.21a	1.32a	0.97b	0.00
2	Nursery establishment	0.00c	1.17a	0.70b	0.00
3	Field preparation	2.42	2.40	2.03	0.08
4	Sowing/Transplanted	1.53b	2.23a	0.59c	0.00
5	Harvesting	1.08	1.18	1.22	0.56
6	Threshing	0.64 ab	0.86a	0.46b	0.01
7	Irrigation	1.91c	3.16b	3.94a	0.00
8	Seed	0.17b	0.17b	0.19a	0.00
9	Compost/FYM	1.27	1.33	1.25	0.63
10	Nitrogen fertilizer	4.86b	5.60a	4.79b	0.00
11	Phosphorus fertilizer	0.27	0.29	0.27	0.15
12	Potassium fertilizer	0.26b	0.31a	0.29 ab	0.00
13	Male labor	1.06b	1.69a	1.03b	0.00
14	Female labor	0.09b	0.22a	0.07b	0.00
15	Herbicide	0.20b	0.11c	0.29a	0.00
16	Insecticide	0.25b	0.32b	0.61a	0.00
17	Fungicide	0.05	0.03	0.05	0.06
18	Rodenticide	0.00	0.01	0.01	0.59
19	Molluscicide	0.00	0.01	0.01	0.16
20	Total grain yield energy (GYE)	62.65c	70.51b	76.75a	0.00
21	External agronomic energy inputs (AEI)	17.28b	22.42a	18.77b	0.00
22	Net energy yield/balance (NEY)	45.37c	48.09b	57.98a	0.00
23	Net energy yield productivity (NEYp) (GJ t^−1^)	10.64b	10.36b	11.37a	0.00

*DSR = dry seeded, PTR = manual puddled transplanted, MTR = machine transplanted rice. Similar letter in the table indicate non-significant difference at p < 0.05 in each row.

### 3.7 Child labor and women empowerment

Rice farmers in Odisha have poor to fair child support, and child labor is considerable in farm activities (Table 3). Total score of child labor showed poor 60%, fair 4% and good 35% for rice cultivation. The tendency to involve children in transplanting activities seems to be higher among smallholder farms. This is indeed a concern and needs to be addressed. Child labor score was lowest for MTR (65), followed by DSR (63) and the highest in PTR (54), indicating MTR and DSR discouraged child labor use in rice cultivation due to more mechanized operations. Promotion of mechanized transplanting or DSR becomes an imperative in the context of child welfare.

Women empowerment was loosely defined in the questionnaire. It was difficult to attribute differences in the scores based on the specific technologies, as the influence is not direct and the causal relationships are not clear. The dimensions used for assessing empowerment are driven by social and cultural norms and economic status of households. So, it is hard to judge whether some technologies are more or less sustainable with regard empowerment. This needs more in-depth research including qualitative methods.

Using the current questionnaire, 80% of the women who responded indicated that empowerment was poor (Table 3). A disaggregated analysis (SI-Table 6) showed that the low scores on this count were a result of low control of women over decisions within collectives/social groups, their personal income, access to information and capacity building for new technologies, decisions regarding household agricultural production and, technology choice (Table 4 and SI-Table 7). This highlights the need to focus on bridging gender gaps in women’s access to knowledge, information, and capacity regarding new technologies. Evidence shows that when equipped with knowledge, information, and capacity women tend to negotiate household decisions better.

However, it was also observed that women’s control over decisions regarding use of household and personal incomes increased with land holding. This is reflected in the overall higher empowerment scores generally observed in households with larger landholdings.

**Fig. 7:**
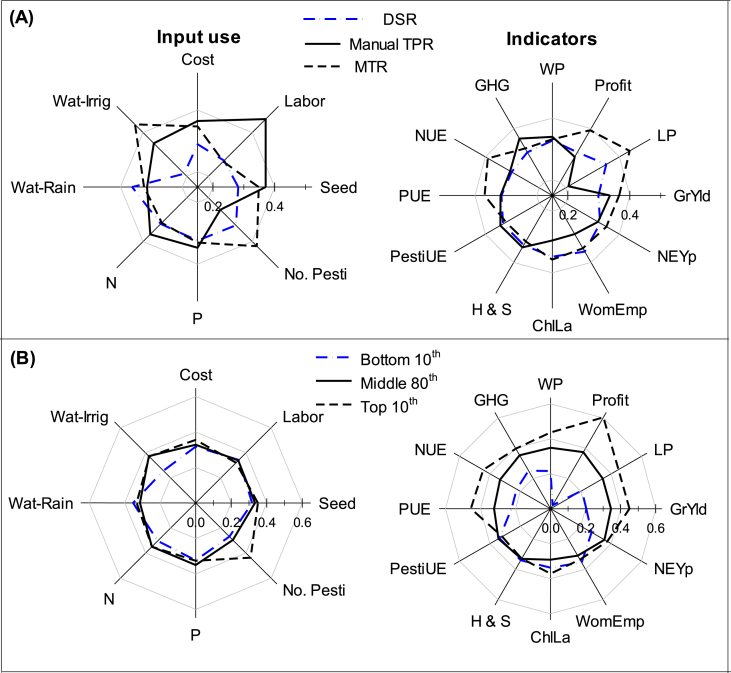
Trade-off comparisons between different production inputs and performance indicators among three different establishment methods and the categories of farmers (delineated by grain yield performance percentile category) in Odisha. The figures on the left side in each panel summarize input use, and the right side summarize the performance indicators. Symbols and units are: Seed = seed input (kg ha^−1^), Labor = labor input (person-days ha ^−1^), Cost = cost ($ ha^−1^), Wat-Irrig = irrigation water input (amount of irrigation, mm season^−1^); Wat-Rain = rainfall (mm season^−1^); N = nitrogen fertilizer input (elemental N, kg ha^−1^), P = phosphorus fertilizer input (elemental P, kg ha^−1^), No. Pesti. = (number of cumulative crop protection product input applications per season). For indicators: GrYld = grain yield (kg ha^−1^), LP = labor productivity (GY Labor^−1^); Profit = profit from rice ($ ha^−1^), WP = water productivity (kg grain L^−1^ water (irrigation + rainfall), GHG = greenhouse gas emission (CO_2_-equivalent kg ha^−1^), NUE = nitrogen use efficiency (kg grain kg^−1^ fertilizer N), PUE = phosphorus use efficiency (kg grain kg^−1^ fertilizer P), PestiUE = crop protection score (using the SRP method), H & S = health and safety, ChiLa = child labor, WomEmp = women empowerment, and NEYp = Net energy productivity.

However, we also observed some trade-offs, where better women empowerment occurred with DSR and MTR (Fig. 7) as manual labor requirement was decreased. This has implications for hired labor and the incomes of men and women thereof. Male family labor increases with the use of machine transplanters (MTR), while the participation of hired labor (both men and women) declines. A significant large decrease is observed in women’s hired labor and this is particularly higher in households with larger land holdings. Higher the availability of machinery and service providers in the village, lower is the participation of hired labor of men and women. While it is important to use DSR and MTR to reduce drudgery of women engaged in the transplanting operations, one also needs to be aware of the trade-offs in terms of potential loss of employment and associated income for the women and men who depend on wage labor. Other means of compensating for that needs to be developed. Engaging women as mechanization service providers is a potential option, if support can be provided to them to engage in such entrepreneurial activity.

### 3.8 Trade-off among input use and performance indicators across three establishment methods and three yield gap categories

The DSR had the lowest seed, labor, production cost, irrigation water application, and N and P fertilizer amount (Table 1; Fig. 7), while PTR had the highest amount of seed, labor, production cost and the amount of N and P fertilizer. Machine transplanted rice was in between for most of the input use except irrigation water and pesticide application. In terms of SRP defined PIs, MTR had the highest yield, net profit, labor productivity, NUE, PUE, child labor and women empowerment, and the lowest in pesticide use efficiency, and health and safety indicators. The top 10th percentile farmers (n = 83, 12.7% farmers) not only applied higher input use (except labor), they also had the highest PIs, i.e., labor productivity, net profitability, NUE, PUE, water productivity, and low child labor use and were at par for health and safety and women empowerment with the middle 80th percentile. However, they had highest production of CO_2_ equivalent and lowest pesticide use efficiency (Fig. 7).

### 3.9 Energy use efficiency

Manual PTR had the highest AEI followed by MTR and the lowest in DSR (by 23% than PTR). Machine transplanted rice had the highest GYE, NEY, and NEYp (Table 5). In terms of energy productivity, PTR was the lowest, and the major energy input difference between DSR and PTR was in field preparation and sowing/transplanting, where DSR had 32% less energy input (3.95 GJ ha^−1^) than PTR (5.8 GJ ha^−1^; Table 5). In all methods, N fertilizer had the highest AEI followed by field preparation and irrigation.

**Fig. 8:**
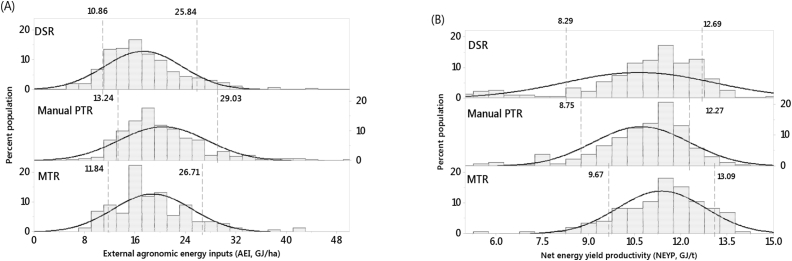
The frequency distribution of external agronomic energy inputs (AEI) (A) and net energy yield productivity (NEYp) (B) for three rice establishment methods in the survey villages Odisha, India, 2016. DSR = dry direct seeded, PTR = manual puddled transplanted, MTR = machine transplanted rice. Fitted line inside the plot indicate normality curve and the two vertical dotted lines are the 10th and 90th percentile and their values for each establishment method.

The frequency distribution of AEI and NEYp (Fig. 8) showed DSR was towards the higher range for AEI, while MTR followed by DSR are more toward higher range in NEYp. Considering all energy related indicators, for the maximization of energy use efficiency and productivity, MTR followed by DSR are the better performing establishment methods.

## 4 Discussion

### 4.1 Sustainability of rice establishment methods

Machine transplanted rice showed the better performance with highest yield, profit, labor productivity, nitrogen-, phosphorus-use efficiency, and water productivity (at par), and the overall energy productivity, indicating it has a high potential to replace manual PTR (Fig. 7). Whereas with DSR, 14% of farmers were mapped in the bottom decile (≤net profit of $81), indicating a high economic risk associated with this approach. The better performance of MTR might be because of synchronization of planting time (Kar et al., 2018), reduced transplanting shock by use of young seedlings (Chen et al., 2017), early seedling vigor and uniform plant stand (Lal and Roy, 1996). Along with better crop performance, MTR also reduces production cost and drudgery, reduces health risks for farm laborers (high labor productivity), especially women and children and offers more opportunities for mechanization in rice establishment as well as better employment and business opportunities for rural youth and women through the development of custom hiring services (Malik et al., 2011). However, the challenges hindering MTR adoption at scale include the fact that it is a relatively new technology which has not been widely tested and verified at the farmers field leading to poor awareness of the benefits of the technology, high initial cost of the machine, limited availability of machines, unavailability of skilled operators, lack of maintenance and troubleshooting facilities, lack of mat-type nursery raising skills, unsuitability of technology for lowland rainfed ecology, and uncertain timing of access to an irrigation facility (Sudhir-Yadav et al., 2017).

Direct seeded rice provided benefits of reducing drudgery, business opportunities for women and youth, and synchronization of planting time, decreased the cultivation cost by more than $100 ha^−1^; and increased labor productivity by more than 20–60 kg day^−1^. However, there were trade-offs of a large yield gap (41%) and having a large percentage of farmers in the bottom decile compared to the other two crop establishment methods (Fig. 2). The yield gap is high compared to previous findings for lowland rice production (Lobell et al., 2009; Stuart et al., 2016). The largest contribution to a yield gap in DSR besides fertilizer and water use, are weeds (Chauhan et al., 2012), poor crop management practices (Devkota et al., 2019b, Devkota et al., 2019a), and specificity of the production packages of DSR (Kumar and Ladha, 2011). To reduce the risks of the DSR-based system, more research is required on improved plant nutrition, water and weed management, suitable seeding machines (durability, cost, access to maintenance), and characterization of suitable areas for DSR. The farmers used less seed with drill seeding than for PTR or MTR. A high seed rate for transplanting is adopted to mitigate the risk of seedling damage (Huang et al., 2018). In spite of high variability in yield, the major drivers for adoption of DSR are labor scarcity, lower input costs, and higher net profit than PTR (Kumar and Ladha, 2011).

Further, DSR and MTR showed a potential for GHG emission reduction. DSR reduced GHG emission by 19% than PTR and by 14% than MTR. Annually, approximately 96.1 M t CO_2_ equivalent emission from the paddy production in India (FAOSTAT, 2019). It showed the potential to reduce CO_2_ emission from rice field by 5.7 M t through MTR and by 13.4 M t by DSR adoption. The lowest CO_2_ equivalent emission from DSR (Fig. 6) came mostly through the implementation of non-puddled preparation of fields (dry-direct seeding), no pre-season flooding, and mostly through the implementation of frequent alternate-wetting and drying (AWD) method of irrigation (Dong et al., 2006). Further, in PTR, farmers often used higher fertilizer rates (Table 1), and higher fossil energy input, especially during crop establishment (Table 5), which is yet to be accounted while computing the GHG emissions. Further, DSR provided option for capturing highest amount of rainfall (15% than manual PTR) mostly due to offering opportunity for early/timely planting; and MTR improved NUE and PUE mostly due to better yield. All these indicated, the environmental sustainability from alternative establishment methods (DSR and MTR) can be better than manual PTR.

This study shows N- and P-use efficiencies (Fig. 5) are limiting yield of DSR. The mean and median N and P use efficiency values for all crop establishment methods were within the optimization zone of NUE 40–80 kg grain kg^−1^ N and PUE 150–300 kg grain kg^−1^ P (Marinus et al., 2016; Devkota et al., 2019b, Devkota et al., 2019a), therefore there is scope for improvement for both N and P use efficiencies. However, the scope for increasing these efficiencies mainly through the optimization of nutrient application (too low application rate could lead to soil nutrient mining, and too high application would be wasteful and may cause negative effects to the environment) also increases the cost of production (Devkota et al., 2019b, Devkota et al., 2019a).

The gold or acceptable range of most of the farmers for pesticide application in rice was reached mostly due to low or no application of pesticides, unlike in other Asian countries. There were more applications of pesticides in MTR and DSR than PTR, and the order of use these pesticides were mostly herbicides, insecticides, and fungicides, respectively. Despite relatively low application of pesticides, farmers in Puri district scored lower value in the health and safety indicator. Farmers were ranked in the poor or fair category, mostly due to poor management of pesticides and did not adopt the appropriate safety measures for example personal protective clothing and masks, did not follow properly the re-entry time into a crop after application of a pesticide, and had inadequate pesticide storage and disposal rules. Training and awareness programs addressing safe handling of pesticide and safety measures as well as education concerning the long-term risks of pesticide exposure on health and the environment has improved the safety behavior of farmers (Bhandari et al., 2018). Such training is imperative in Odisha.

The approach of this study was not sufficient to provide detailed measures of women empowerment and child labor. Nevertheless, this study indicated that MTR and DSR decreased child labor. Women farmers indicated that these technologies are time- and cost-saving, reduce drudgery, and they improved crop establishment and yield. This is consistent with findings of previous studies in rice systems (Akter et al., 2017; Ganguly et al., 2015). However, the major challenges associated with the technologies are the women’s lack of experience in handling them and challenges with the operational details involved in their use. This reflects that rural women in Puri district have not had the same opportunities to attend training as men, as indicated by the low scores on their access to information and capacity development activities in relation to the new technologies.

### 4.2 Comparison of sustainability indicator gaps across establishment methods and different yield category farmers

In Odisha, there is significant scope for rice production to be more sustainable economically, environmentally and socially. A key finding that supports this contention is the rather high attainable (exploitable) yield gap of 1.52 t ha^−1^; a high yield gap when compared to lowland rice production in six other Asian countries (Stuart et al., 2016). If we compare the same yield gap category of farmer, there was a profit gap of $273 ha^−1^, labor productivity gap of 21 kg labor^−1^, NUE gap of 22 kg grain kg^−1^ N, PUE gap of 105 kg^−1^ P. Even the top 10 decile farmers application of N (82 kg N ha^−1^) and P fertilizer rates (19 kg P ha^−1^) are below the state recommended rate of 80–100:40:40 kg N:P_2_O_5_:K_2_O ha^−1^ (RKMP, 2018). This is even more of a concern when the current fertilizer practices provide low use efficiencies (Fig. 5). In Odisha, it is both the sub-optimal amount of fertilizer applied and the fertilizer use efficiencies of applied fertilizers that limiting the yield of rice. Further, the water productivity and the amount of irrigation and rainfall water in rice indicated that the majority of the rice was produced under what was in effect rainfed condition. Yield variability is heavily controlled by fertilizer use, irrigation, and climate and the productivity can be increased by closing yield gaps through changes in fertilizer and irrigation management practices (Mueller et al., 2012).

Overall the performance indicators worked quite well in assessing the trade-offs with different technologies; however, there is significant room for improvement in these indicators versus scale of assessment: while most of the indicators worked well to evaluate the technologies at plot scale, clearly some of the indicators e.g. food and health safety, child labor and women empowerment are not always technology or field specific. Some of these are more appropriate to be measured at the regional level while others may work at the household level. Sustainable Rice Platform performance indicators (version 1.0) are not structured well at this stage to dissect the difference of technological intervention in terms of food safety. As food safety is more a regional/household indicator, most of the farmers responded generically irrespective of technology. This indicator could be computed in a better way via more explanatory questioning related to food safety; avoiding laboratory analysis could make the indicator computation simple. Similar to food security, performance indicators for health and safety are more relevant at the household level. Most of the respondents were sharing information based on their practices on their farm rather than for a specific technology.

The computed energy input in different operations (Table 5) and the use efficiencies, i.e., energy input (AEI), energy output (GYE), energy balance (NEY), and net energy productivity (NEYp) indicators for transplanted rice is in consistent with values by Quilty et al. (2014) and Eskandari and Attar (2015). Machine transplanted rice had highest GYE, NEY, and NEYp while manual PTR had highest AEI, which suggests better sustainability of MTR than PTR.

### 4.3 Suggestions for further refinement of sustainability indicators

We suggest the following considerations and refinements to the measurement of the performance indicators for sustainable rice cultivation:

1. The household in this study were farmers. It was relatively easy for the farmers to respond to questions on their current practices, but they found it difficult to respond to indicators which are more at the landscape level (e.g. heavy metal contamination, type of contamination, etc).
2. The application of rodenticide, molluscicide and avicide are sporadic and minimal. Further, the ingredients could be many fold more toxic than the molecules of herbicides and fungicides. So, while computing pesticide use efficiencies, consideration of the LD50 rate of the pesticide could make the computed value more convincing. Computation of the pesticide indicator may focus only on the applied pesticides (for example if avicides are not applied by any farmers in the surveyed areas, this pesticide category can be omitted from the computation) or use computations that consider how much active ingredient is applied from the most commonly applied pesticides: herbicides, insecticides, and fungicides.
3. Computing water productivity in terms of L of water per kg of grain would provide an easier index for practitioners and extensionists to understand, than an indicator expressed as kg of grain per L of water.
4. Similarly, some expressions such as high GHG score are misleading to assess the sustainability performance of rice production practices.
5. Women’s empowerment in households is driven by prevailing social and cultural norms and other intersectional factors. They are not technology-specific. In fact, technology use by women and their ability to make decisions regarding them is influenced by their empowerment status. While reduction in labor use and associated drudgery is welcome, some poor women who depend on wage labor for transplanting might also lose employment and incomes due to mechanization. This might open up opportunities for some entrepreneurial women to engage in service provision role. The current indicators do not allow capturing such tradeoffs in a technology-specific context. While the use of structured questionnaire and indicators is helpful, much insights would be gained from using mixed methods for such assessments.
6. Indicators of energy use can be expressed using different indicators, and the outputs can be confusing for extension specialists and farmers. For example, GEY, AEI, NEY, NEYp, differ with different technologies via the input use, machines use, time and intensity of field operations, and the final outputs (grain and straw). We contend that because NEYp is more representative, then the inclusion of NEYp as the 13th performance indicator of sustainable rice cultivation could add value through providing a clearer message than the other measures, especially for technology comparisons and impact evaluation.

## 5 Conclusions

The differential trade-off among different SRP PIs with different rice establishment methods in Odisha indicated MTR is the best performed has advantage over other establishment methods considering its economic, environmental and social indicators performmance as defined by SRP. Further, a relatively high gap (target to achieve) in yield (41%) and similar gaps in profit, labor productivity, NUE, PUE, and water productivity indicated sustainability can further be improved. Opportunities were identified to both decrease the yield gap and increase profitability by optimizing N and P fertilizer use, irrigation, pesticides and labor. Such optimization is crucial to reduce the nutrient mining and improve the land productivity. Our findings clearly show the benefits and positive potential (reduce sustainability trade-off) of MTR in Odisha as an alternative to PTR. Machine transplanted rice provided higher yield and net profit, and better performance of most of the SRP indicators and measures of energy use efficiency. There is scope for reducing the environmental footprint by reducing GHG emissions through the adoption of DSR. MTR-technology appeared to be the best in terms of increasing water productivity, NUE and PUE. The highest labor productivity in MTR and DSR suggests the opportunity of mechanization in rice production using alternative establishment methods and thus improving labor productivity. Machine transplanted rice also reduces drudgery and child labor, and therefore opens potential new income generation opportunities through women and youth engagement as entrepreneurial service providers. Except for food safety and workers health and safety, which turned out to be more watershed and household level indicators, SRP defined PIs (version 1.0) and the gap approach provide an effective set to measure the sustainability (economic, environmental and social) of different technologies, and provided the target value to achieve for establishing sustainable rice production and sustainable intensification.

## Footnotes

Appendix A Supplementary data to this article can be found online at https://doi.org/10.1016/j.jclepro.2019.118835.
