# Supplementary material for: Assessing alternative crop establishment methods with a sustainability lens in rice production systems of Eastern India
Source: J Clean Prod. 2020 Jan 20;244:118835. doi: 10.1016/j.jclepro.2019.118835 (PMC6946438; doi:10.1016/j.jclepro.2019.118835)
Supplement: Multimedia component 1 [file mmc1.docx]

**Supplementary information (SI)**

Table 1. Performance indicators (PIs) for sustainable rice cultivation as defined by the Sustainable Rice Platform (SRP) standards, 2015.

| Indicator No. | Indicator name | Category | Guiding principles |
| --- | --- | --- | --- |
| 1 | Profitability: net income from rice | Economic | Improved livelihoods |
| 2 | Labor productivity | Social | Improved livelihoods |
| 3 | Productivity: grain yield | Economic | Improved livelihoods |
| 4 | Food safety | Social | Consumer needs |
| 5 | Water-use efficiency | Environmental | Resource use efficiency and sustainable intensification |
| 6 | Nitrogen use efficiency | Environmental | Resource use efficiency and sustainable intensification |
| 7 | Phosphorus use efficiency | Environmental | Resource use efficiency and sustainable intensification |
| 8 | Pesticide use efficiency | Environmental | Resource use efficiency and sustainable intensification |
| 9 | Greenhouse gas emission | Environmental | Climate change mitigation |
| 10 | Health and safety | Social | Labor conditions |
| 11 | Child labor | Social | Labor conditions |
| 12 | Women empowerment | Social | Social development |

Table 2. Energy equivalent conversion factors for the computation of agronomic inputs and outputs from rice grain yield.

| SN | Agronomic input | MJ unit^−1^ | References |
| --- | --- | --- | --- |
| 1 | Diesel (L^−1^) | 44.8 | Bertrand et al. ( 2016) |
| 2 | Irrigation (m^-3^) | 1.02 | Rafiee et al. (2010) |
| 3 | Seed (kg^−1^) | 15.5 | Quilty et al. (2014) |
| 4 | Compost/FYM (dry wt. kg^−1^) | 0.3 | Singh and Mittal (1992) |
| 5 | Nitrogen (kg^−1^) | 63.5 | Pimentel and Pimentel (2007) |
| 6 | Phosphorous (kg^−1^) | 13.95 | Pimentel and Pimentel (2007) |
| 7 | Potassium (kg^−1^) | 6.69 | Pimentel and Pimentel (2007) |
| 8 | Male labor (h^-1^) | 1.96 | Pishgar-Komleh et al. (2011) |
| 9 | Female labor (h^-1^) | 1.57 | Pishgar-Komleh et al. (2011) |
| 10 | Herbicide (kg^−1^) | 303.8 | Pimentel and Pimentel (2007) |
| 11 | Insecticide (kg^−1^) | 418.4 | Pimentel and Pimentel (2007) |
| 12 | Fungicides (kg^−1^) | 115b | Pimentel and Pimentel (2007) |
| 13 | Rodenticides (kg^−1^) | 255 | Pimental et al. (1993) |
| 14 | Molluscicides (kg^−1^) | 205 | Pimental et al. (1993) |
| 15 | Rice grain yield (kg^−1^) | 15.2 | Pimentel and Pimentel (2007) |
| 16 | Animal power (h^-1^) | 14.6 | Quilty et al. (2014) |

Table 3. Percentage of farmers using, one application, two applications and three applications of different category pesticides for controlling weeds, insects, diseases, and rats in Odisha, India, 2016.

| Establishment method | Pesticide category | No. of farmers (% of total) | No. of farmers with one application (% of those who used pesticides) | No. of farmers with two applications (% of those who used pesticides) | No. of farmers with three applications (% of those who used pesticides) |
| --- | --- | --- | --- | --- | --- |
| DSR | Herbicide | 179 (72) | 161 (90) | 17 (10) | 1 (0) |
| (N=250) | Insecticide | 178 (71) | 164 (92) | 13 (8) | 1 (0) |
|  | Fungicide | 89 (36) | 84 (94) | 5 (6) | 0 |
|  | Rodenticide | 6 (2) | 6 (100) | 0 | 0 |
| Manual PTR | Herbicide | 128 (49) | 124 (97) | 4 (3) | 0 |
| N=260 | Insecticide | 143 (55) | 125 (87) | 17 (13) | 1 (1) |
|  | Fungicide | 90 (35) | 88 (98) | 2 (2) | 0 |
|  | Rodenticide | 13 (5) | 13 (100) | 0 | 0 |
| MTR | Herbicide | 115 (81) | 109 (95) | 5 (4) | 1 (1) |
| N=142 | Insecticide | 114 (80) | 86 (75) | 28 (25) | 0 |
|  | Fungicide | 78 (55) | 68 (87) | 10 (13) | 0 |
|  | Rodenticide | 8 (6) | 8 (100) | 0 | 0 |
| Total | Herbicide | 422 (67) | 394 (93) | 26 (6) | 2 (1) |
| (N=652) | Insecticide | 435 (69) | 375 (86) | 58 (13) | 2 (1) |
|  | Fungicide | 257 (42) | 240 (93) | 17 (7) | 0 |
|  | Rodenticide | 27 (4) | 27 (100) | 0 | 0 |

Table 4. Farmers’ averaged scores on health and safety indicator based on SRP scorecard for different rice establishment methods in Odisha, India, 2016. Values are mean + SD. Each question had the maximum score of 10. DSR = dry seeded, PTR = manual puddled transplanted, MTR = machine transplanted rice

| SN | Health and safety rules and regulations | DSR | Manual transplanting (PTR) | MTR | Overall |
| --- | --- | --- | --- | --- | --- |
| 1 | Incidence of work-related accident | 7.64 + 4.23 | 7.77 + 4.10 | 7.01 + 4.42 | 7.55 + 4.23 |
| 2 | Safety instruction and first aid | 2.92 + 4.56 | 2.65 + 4.42 | 3.45 + 4.77 | 2.93 + 4.55 |
| 3 | Calibration of the spray equipment | 5.28 + 1.59 | 3.71 + 2.63 | 5.32 + 2.25 | 4.66 + 2.33 |
| 4 | Training pesticide applicators | 3.88 + 4.24 | 5.37 + 4.43 | 5.00 + 4.73 | 4.72 + 4.47 |
| 5 | Use of personal protective equipment | 6.82 + 3.50 | 7.46 + 3.66 | 5.49 + 3.69 | 6.79 + 3.68 |
| 6 | Washing and changing facilities | 0.00 + 0.00 | 0.00 + 0.00 | 0.00 + 0.00 | 0.00 + 0.00 |
| 7 | Applicators restrictions | 4.01 + 2.51 | 5.48 + 3.39 | 3.69 + 2.09 | 4.53 + 2.93 |
| 8 | Re-entry time | 5.18 + 3.92 | 6.67 + 4.00 | 4.47 + 3.66 | 5.62 + 3.99 |
| 9 | Pesticide storage | 8.90 + 2.43 | 9.04 + 2.41 | 7.99 + 2.92 | 8.76 + 2.57 |
| 10 | Pesticide disposal | 6.28 + 4.84 | 7.38 + 4.40 | 5.92 + 4.93 | 6.64 + 4.73 |

Table 5. Farmers’ response (%) to individual questions based on SRP scorecard for health and safety indicator for rice production (averaged of three establishment methods) as defined by SRP in Odisha, India, 2016.

| SN | Requirements | Level of performance (Please tick in one answer in each question) | | Response (%) | |  |
| --- | --- | --- | --- | --- | --- | --- |
| 1 | Incidence of work related accidents | No | | 69.4 | |  |
|  |  | 1. No minor or major work-related injuries or ill health | | 18.4 | |  |
|  |  | 1. No major work-related injuries or ill health, but minor cases in a lower frequency than in the last crop cycle | | 11.9 | |  |
|  |  | 1. Any major work-related injuries or minor cases in an equal or higher frequency than in the last crop cycle | | 0.3 | |  |
| 2 | Do you have first aid box? | No | | 68.6 | |  |
|  |  | 1. first aid supplies are available on-farm | | 0.5 | |  |
|  |  | 1. first aid supplies are available at house | | 24.2 | |  |
|  |  | 1. At both places (farm and house) | | 3.7 | |  |
|  |  | 1. There is no first aid supplies are available on-farm | | 3.0 | |  |
| 3 | Calibration | 1. Calibration and maintenance within current crop cycle | | 8.7 | |  |
|  |  | 1. Calibration and maintenance within the past 2 years | | 0.9 | |  |
|  |  | 1. No Calibration and maintenance within the past 2 years | | 2.6 | |  |
|  |  | 1. Outsource the service | | 87.8 | |  |
| 4 | Training to the pesticide applicators | 1. There is no use of pesticides | | 21.0 | |  |
|  |  | 1. Pesticide applicators participated in a training session in the past 1 years | | 15.7 | |  |
|  |  | 1. Pesticide applicators participated in a training session in the past 3 year | | 19.7 | |  |
|  |  | 1. Pesticide applicators participated in a training session in the past 5 years | | 43.3 | |  |
|  |  | 1. Pesticide applicators did not participate in a training session in the past 5 year | | 0.3 | |  |
| 5 | Personal protective equipment | 1. There is no use of pesticide | | 21.2 | |  |
|  |  | 1. Pesticide applicators use at least three among (glove, mask, boots, protective clothing), but always use gloves of good quality, and clothing is washed after use | | 27.7 | |  |
|  |  | 1. Pesticide applicator use at least two (gloves, masks, boots, protective clothing), but always gloves of good quality, and clothing is washed after use | | 33.8 | |  |
|  |  | | 1. Pesticide applicators use fewer than two of the four items, or do not use gloves, or use items of low quality, or clothing is not washed after use | | 17.4 | |
| 6 | Applicator restrictions | 1. There is no use of pesticide | | 21.2 | |  |
|  |  | 1. Pesticides are not applied by pregnant or lactating women or by children below 18 years age, or by person who suffer from chronic or respiratory diseases | | 77.7 | |  |
|  |  | 1. Pesticides are applied by pregnant or lactating women or children below 18 years, or by person who suffer from chronic or respiratory diseases | | 1.1 | |  |
| 7 | Re-entry time | 1. There is no use of pesticide | | 21.2 | |  |
|  |  | 1. The recommendation, or re-entry after 48 hours is observed and communicated by placing warning signs in the field | | 16.0 | |  |
|  |  | 1. The recommendation, or re-entry after 48 hours is observed and communicated verbally | | 34.9 | |  |
|  |  | 1. The recommendation, or re-entry after 48 hours is not observed or not communicated | | 25.0 | |  |
| 8 | Pesticide storage | 1. There is no use of pesticide or inorganic fertilizers | | 21.2 | |  |
|  |  | 1. Pesticides and inorganic fertilizers are labeled and stored in a locked and separate place | | 57.4 | |  |
|  |  | 1. Pesticide and inorganic fertilizers are labeled and stored in a general farm storage area | | 17.1 | |  |
|  |  | 1. Pesticides and inorganic fertilizers are not labeled or stored | | 4.4 | |  |
| 9 | Pesticide disposal | 1. There is no use of pesticide | | 20.8 | |  |
|  |  | 1. Farmer participate in a collection, return, or disposal system | | 3.3 | |  |
|  |  | 1. Empty containers are rinsed three times with water and made unusable by crushing or puncturing before burying them on the farm and are not recycled | | 45.4 | |  |
|  |  | 1. Empty container are rinsed and re used. | | 0.3 | |  |
|  |  | 1. Surplus spray and wash water is applied over an unmanaged part of the farm, away from water bodies | | 22.8 | |  |
|  |  | 1. Obsolete pesticides (date expired and banned) are returned to dealers or, if not possible, disposed of in a manner that minimize exposure to humans and environment | | 4.3 | |  |
|  |  | 1. There is a collection, return, or disposal system, but it is not used. In the absence of such a system, empty pesticide containers and obsolete pesticides are not disposed as explained above | | 3.3 | |  |

Table 6. Percent farmers’ response regarding women empowerment in rice production as defined by SRP standards in Odisha, India, 2016.

| SN | Requirements | Level of performance (Please tick in one answer in each question) | Response (%) |
| --- | --- | --- | --- |
| 1. | Women's control over decisions regarding household agricultural production | 1. Women have at least equivalent decision-making power | 19.5 |
|  |  | 1. Women have some but less than equivalent decision-making power | 35.9 |
|  |  | 1. Women have no or marginal decision-making power | 44.5 |
| 2. | Women's role and control over decision regarding selected technology | 1. Updated/familiar on adoption of new technology | 60.7 |
|  |  | 1. Not updated/familiar on adoption of new technology | 39.3 |
| 3. | What has changed for women with new technology | 1. Decision-making power increased | 7.2 |
|  |  | 1. Decision-making power decreased | 28.8 |
|  |  | 1. Their own labor input decreased | 61.1 |
|  |  | 1. Their own labor input increased | 2.8 |
| 4. | Women's satisfaction regarding new technology | 1. Not involved | 24.0 |
|  |  | 1. Good for increasing income | 40.2 |
|  |  | 1. Good for men but not for women | 11.2 |
|  |  | 1. Good for both men & women | 22.9 |
|  |  | 1. Hazardous |  |
| 5. | Women's satisfaction regarding their labor input | 1. Women are satisfied | 54.5 |
|  |  | 1. Women are partly satisfied (e.g., no balance during peak labor-requirement periods) | 31.3 |
|  |  | 1. Women are unsatisfied | 14.2 |
| 6. | Women's access to information and capacity building for new technology (in broader context) | 1. Women have equal access | 16.2 |
|  |  | 1. Women have less access | 30.9 |
|  |  | 1. Women have no access | 52.9 |
| 7. | Women's access to seasonal resources for farm activities | 1. Women have at least equivalent decision-making power and equal access | 18.6 |
|  |  | 1. Women have some but less than equivalent decision-making power and less than equivalent access | 24.1 |
|  |  | 1. Women have no or marginal decision-making power and no access | 57.3 |
| 8. | Women's control over decision-making regarding household income | 1. Women have at least equivalent decision-making power | 23.7 |
|  |  | 1. Women have some but less than equivalent decision-making power | 19.6 |
|  |  | 1. Women have no or marginal decision-making power | 56.5 |
| 9. | Women's control over their personal income | 1. Women have equivalent or greater control | 16.8 |
|  |  | 1. Women have some but less than equivalent control | 26.0 |
|  |  | 1. Women have no or very limited control | 57.2 |
| 10. | Women's participation in collective decision-making | 1. Women participation in group leadership, are active in group decision, and their voices are valued | 11.0 |
|  |  | 1. Women are present during group decision, but their contributions are not given full weight | 49.5 |
|  |  | 1. Women are excluded from group decision-making | 39.5 |
| 11. | Violence against women | 1. There are no cases of violence | 96.4 |
|  |  | 1. There is at least one case of violence | 3.6 |

**References**

Bertrand, E., Pradel, M., Dussap, C.-G., 2016. Economic and Environmental Aspects of Biofuels, in: Green Fuels Technology. Springer, pp. 525–555.

Pimentel, D., McLaughlin, L., Zepp, A., Lakitan, B., Kraus, T., Kleinman, P., Vancini, F., Roach, W.J., Graap, E., Keeton, W.S., Selig, G., 1993. Environmental and economic effects of reducing pesticide use in agriculture. Agric. Ecosyst. Environ. 46, 273–288. https://doi.org/https://doi.org/10.1016/0167-8809(93)90030-S

Pimentel, D., Pimentel, M.H., 2007. Food, energy, and society, 3rd ed. CRC Press Taylor & Francis Group 6000 Broken Sound Parkway NW, Suite 300 Boca Raton, FL 33487-2742. https://doi.org/https://doi.org/10.1201/9781420046687

Pishgar-Komleh, S.H., Sefeedpari, P., Rafiee, S., 2011. Energy and economic analysis of rice production under different farm levels in Guilan province of Iran. Energy 36, 5824–5831. https://doi.org/https://doi.org/10.1016/j.energy.2011.08.044

Quilty, J.R., McKinley, J., Pede, V.O., Buresh, R.J., Correa Jr, T.Q., Sandro, J.M., 2014. Energy efficiency of rice production in farmers’ fields and intensively cropped research fields in the Philippines. F. Crop. Res. 168, 8–18. https://doi.org/https://doi.org/10.1016/j.fcr.2014.08.001

Rafiee, S., Avval, S.H.M., Mohammadi, A., 2010. Modeling and sensitivity analysis of energy inputs for apple production in Iran. Energy 35, 3301–3306.

Singh, S., Mittal, J.P., 1992. Energy in production agriculture. First Edition, Mittal Publications, New Delhi, 166pp.

**Appendix 1.**

**Household questionnaire used for the sustainability assessment of three rice establishment methods in Odisha, India, 2016**

Name of Enumerator_______________________Targeted Technology_______________

1. **General Information**

| Country: | India | Year | 2016 |
| --- | --- | --- | --- |
| Data Source: | Field survey | Season | Wet season, 2015 |

1. **Respondent’s details**

| Name: |  | | District: | Puri |
| --- | --- | --- | --- | --- |
| Father’s name: |  | | Block: |  |
| Mobile: |  | | GP: |  |
| Age: |  | | Village: |  |
| Gender: | Male- | Female- |  |  |
| Education: | Up to Class 5 | Class 5-10 | Class 11-12 | Graduate (BA/MA/PhD) |
| No. of family members: |  | Male- | Female- | Children (Boy and Girl)- |

**Agricultural background**

| Total land holding of the farmer: | in local unit: | | . . . . . . . acre | | | | | | Land holding in (ha): | | |
| --- | --- | --- | --- | --- | --- | --- | --- | --- | --- | --- | --- |
| Land tenure of total land holding: (acre) | Own | | Contract/Share cropping | | | | | | Rented | | |
| Total rice cultivated area: | in local unit: | | . . . . . . . acre | | | | | | Land holding in (ha): | | |
| Cropping pattern: |  | | | | | | | | | | |
| Total number of patches of rice cultivated parcels: |  | | | | | | | | | | |
| Technology adoption/ practiced by the farmer: | Dry seeded rice (DSR) | Machine transplanting (MTR) | | | | Manual transplanting (Random) | | Manual transplanting (Line) | | | Broadcasting |
| Selected Technology: | DSR | Machine transplanting (MTR) | | | | | | Manual transplanting (Random) | | | |
| Source of knowledge for selected technology: | Neighboring farmers | CSISA project | | DOA | | | NGOs | OUAT | | Others | |
| No. of machines in this village for selected technology: | | | | |  | | | | | | |
| No. of service providers in this village for selected technology: | | | | |  | | | | | | |

**1.Indicator-I Productivity, gross income and seed management.**

| **Questions** | | | | | | | | | | **Respondent’s reply** | | | | | | | | | | | | | | |
| --- | --- | --- | --- | --- | --- | --- | --- | --- | --- | --- | --- | --- | --- | --- | --- | --- | --- | --- | --- | --- | --- | --- | --- | --- |
| **Talk about a patch/parcel where farmer got maximum yield from selected technology:** | | | | | | | | | | | | | | | | | | | | | | | | |
| Rice area of selected patch: | | | | | | | | | | In local unit..... | | | | | | | | | . . . . . . acre | | . . . . . . ha | | | |
| Rice variety in this parcel: | | | | | | | | | |  | | | | | | | | | | | | | | |
| Nursery establishment date (dd/mm/yyyy): | | | | | | | | | | Month | | | | Wk-1 | | | | | Wk-2 | Wk-3 | | | Wk-4 | |
|  |  |  |  |  |  |  |  |  |  |  | | | |  | | | | |  |  | | |  | |
| Rice transplanting or sowing date (dd/mm/yyyy): | | | | | | | | | | Month | | | | Wk-1 | | | | Wk-2 | | Wk-3 | | | Wk-4 | |
|  |  |  |  |  |  |  |  |  |  |  | | | |  | | | |  | |  | | |  | |
| Rice emergence date (for DSR/broadcasting): | | | | | | | | | | Month | | | | Wk-1 | | | | Wk-2 | | Wk-3 | | | Wk-4 | |
|  |  |  |  |  |  |  |  |  |  |  | | | |  | | | |  | |  | | |  | |
| Rice harvesting date (dd/mm/yyyy): | | | | | | | | | | Month | | | | Wk-1 | | | | Wk-2 | | Wk-3 | | | Wk-4 | |
|  |  |  |  |  |  |  |  |  |  |  | | | |  | | | |  | |  | | |  | |
| Rice threshing date (dd/mm/yyyy): | | | | | | | | | | Month | | | | Wk-1 | | | | Wk-2 | | Wk-3 | | | Wk-4 | |
|  |  |  |  |  |  |  |  |  |  |  | | | |  | | | |  | |  | | |  | |
| Total fresh grain yield (kg): | | | | | | | | | |  | | | | | | | | | | | | | | |
| Grain price (per kg): | | | | | | | | | |  | | | | | | | | | | | | | | |
| Exchange rate ($1): | | | | | | | | | | $1= INR 65 | | | | | | | | | | | | | | |
| Seed rate used in the patch/parcel (kg): | | | | | | | | | |  | | | | | | | | | | | | | | |
| Seed type: | | | Hybrid | | | Registered | | | Certified | | | Farmer's own | | | | Farmers exchange | | | | | | Others | | |
| Seed price (per kg): | | | | | |  | | | | | | | | | | | | | | | | | | |
| Seed source: | | Own Harvest | | | Exchange  /Co-farmers | | | Seed grower | | Input dealers | | | DOA | | | | Private companies | | | Land owner's | | | | Others |
| Seed treatment: | | | | If yes, seed treatment with | | | | | | | | | | | | | | | | | | | | |
| Yes | No | | | 1. Water | | | 2.Insecticide | | | | 3. Fungicide | | | | 4. Chemical (name not known) | | | | | | | | | |

Existing technologies for the different field operations in the rice production in the surveyed parcel

| **SN.** | **Questions** | | | | | | | | | | | | | | | | | | **Respondent’s reply** | | | | | | | | | | | | | | | | | | | | | | | | | | |
| --- | --- | --- | --- | --- | --- | --- | --- | --- | --- | --- | --- | --- | --- | --- | --- | --- | --- | --- | --- | --- | --- | --- | --- | --- | --- | --- | --- | --- | --- | --- | --- | --- | --- | --- | --- | --- | --- | --- | --- | --- | --- | --- | --- | --- | --- |
| 1 | Laser land leveling: | | | | Yes,-same year | | | | | | | | | | Yes,-Last year | | | | | | | | | | Yes,-2 years back | | | | | | | | | | | | | | Never | | | | | | |
| 2 | Pre-season activities like field cleaning, stones removal, residue burning: | | | | | | | | | | | | | | | | | | Yes | | | | | | | | | | | | | | | | | | | No | | | | | | | |
| 3 | Cleaning and repairing of bunds: | | | | | | | | | | | | | | | | | | Yes | | | | | | | | | | | | | | | | | | | No | | | | | | | |
| 4 | Channel maintenance: | | | | | | | | | | | | | | | | | | Yes | | | | | | | | | | | | | | | | | | | No | | | | | | | |
| 5 | First ploughing: | | | | | Animal | | | | | | | | | | | | | 2-wheel tractor | | | | | | | | | | | | | | | | | | | 4-wheel tractor | | | | | | | |
|  | Attachment: *Rotovator/ Disk harrow/ Tine harrow/Specify if other* | | | | | | | | | | | | | | | | | | | | | | | | | | | | | | | | | | | | | | | | | | | | |
| 6 | First harrowing: | | | | | None | | | | | | Animal | | | | | | | | | | | 2W Tractor | | | | | | | | | | | | 4W Tractor | | | | | | | | | | |
|  | Attachment: *Rotovator/ Disk harrow/ Tine harrow/Specify if other* | | | | | | | | | | | | | | | | | | | | | | | | | | | | | | | | | | | | | | | | | | | | |
| 7 | Second harrowing: | | | | | None | | | Animal | | | | | | | | | | | | | | 2W Tractor | | | | | | | | | | | | 4W Tractor | | | | | | | | | | |
|  | Attachment: *Rotovator/ Disk harrow/ Tine harrow/Specify if other* | | | | | | | | | | | | | | | | | | | | | | | | | | | | | | | | | | | | | | | | | | | | |
| 8 | Rotovation: | | | | | None | | | Animal | | | | | | | | | | | | | | 2W Tractor | | | | | | | | | | | | 4W Tractor | | | | | | | | | | |
|  | Attachment: *Rotovator/ Disk harrow/ Tine harrow/Specify if other* | | | | | | | | | | | | | | | | | | | | | | | | | | | | | | | | | | | | | | | | | | | | |
| 9 | Levelling: | | | | | None | | | Animal | | | | | | | | | | | | | | 2W Tractor | | | | | | | | | | | | 4W Tractor | | | | | | | | | | |
|  | Attachment: *Computer assisted LLL/ Wooden planker/Specify if other* | | | | | | | | | | | | | | | | | | | | | | | | | | | | | | | | | | | | | | | | | | | | |
| 10 | Puddling& planking: | | | | | None | | | Human | | | | | Animal | | | | | | | | | 2W Tractor | | | | | | | | | | | | 4W Tractor | | | | | | | | | | |
|  | Attachment:*Rotovator/ Disk harrow/ Tine harrow/Power tiller/Specify if other* | | | | | | | | | | | | | | | | | | | | | | | | | | | | | | | | | | | | | | | | | | | | |
| 11 | Machine used for DSR: | Bullock drawn seed drill | | | | | | | | 2 W Tractor drawn seed drill | | | | | | | | | | | 4W tractor drawn seed drill | | | | | | | | | | Multi-crop zero-tillage machine | | | | | | | | | | | | | | Others |
| 12 | Machine used for MTR: | Walk behind (Kubota) | | | | | | | | Walk behind (Other) | | | | | | | | Riding type (VST) | | | | | | | | | | Riding type (Others) | | | | | | | | | | | | | | Others | | | |
| 13 | Mat raising: | | | | | | | | | | | | | | | | On plastic sheet | | | | | | | | | | | | On tray | | | | | | | | | | | | | | | | |
| 14 | Transport of seedlings: | | | | | | | | | | | | | | | | Yes | | | | | | | | | | | | No | | | | | | | | | | | | | | | | |
| 15 | Distribution of seedlings to different location: | | | | | | | | | | | | | | | | Yes | | | | | | | | | | | | No | | | | | | | | | | | | | | | | |
| 16 | Transplanting: | | | | | | | | | | | | | | | | Yes | | | | | | | | | | | | No | | | | | | | | | | | | | | | | |
| 17 | Thinning: | | | | | | | | | | | | | | | | Yes | | | | | | | | | | | | No | | | | | | | | | | | | | | | | |
| 18 | Gap filling in DSR/ transplanted rice: | | | | | | | | | | | | | | | | Yes | | | | | | | | | | | | No | | | | | | | | | | | | | | | | |
| 19 | Re-seeding in direct seeded rice: | | | | | | | | | | | | | | | | Yes | | | | | | | | | | | | No | | | | | | | | | | | | | | | | |
| 20 | Fertilizer application method or dose: | | | Farmers own experience | | | | | | | Neighboring friend's recommendation | | | | | | | | | | | Government recommendation | | | | | | | | | | Rice Crop Manager | | | | | | | | | | | | No application | |
| 21 | Irrigation source: | | | | | | | | | | | | | | | | | | | Ground water | | | | | | | | | | | | | | | | Surface water | | | | | | | | | |
| 22 | Irrigation method: | | | | | | | AWD | | | | | | | | | | | | Non-AWD | | | | | | | | | | | | | | Rainfed | | | | | | | | | | | |
| 23 | Pesticide application method: | | | | | | Back pack Knapsac spray | | | | | | | | | | | | | Power spray | | | | | | | | | | Boom spray | | | | | | | | | | Not sprayed | | | | | |
| 24 | Growth regulator application: | | | | | | Plant growth hormones | | | | | | | | | | | | | Micro-nutrients | | | | | | | | | | | | | All of the above | | | | | | | | | | None of the above | | |
| 25 | Method of harvesting: | | | | | | Combine Harvester | | | | | | | | | | | | | Reaper | | | | | | | Manual Harvesting | | | | | | | | | | | | | | Other | | | | |
| 26 | Method of drying: | | | | | | Sun drying | | | | | | | | | Solar dryer | | | | | | | | | | Electric dryer | | | | | | | | | | | | | | | None | | | | |
| 27 | Straw management: | | Combine harvesting of rice and all straw burning after rice harvest | | | | | | | | | | Combine harvesting of rice and all straw left in the field and incorporated | | | | | | | | | | | Manual or combine harvesting and 100% straw removal for animal feeding | | | | | | | | | | | | | Manual or combine harvesting and 50% (20 cm standing) straw retention | | | | | | | | |

**2. Indicator II and III economic profitability and labor productivity. All in national currency, e.g., INR.**

**Land rent:** - - - - - - - - - - for this rice season for the surveyed parcel.

| SN | Activities | Input & other costs | Machinery & transport cost | No. of male labors | | No. of female labors | | Male labor wage rate | Female labor wage rate |
| --- | --- | --- | --- | --- | --- | --- | --- | --- | --- |
|  |  |  |  | Family | Hired | Family | Hired |  |  |
| 1 | Seed and Nursery preparation: |  |  |  |  |  |  |  |  |
| 2 | Compost/FYM: |  |  |  |  |  |  |  |  |
| 3 | Land preparation: |  |  |  |  |  |  |  |  |
| 4 | Transplanting or sowing: |  |  |  |  |  |  |  |  |
| 5 | Herbicide or weeding: |  |  |  |  |  |  |  |  |
| 6 | Fertilizer (All): |  |  |  |  |  |  |  |  |
| 7 | Irrigation: |  |  |  |  |  |  |  |  |
| 8 | Pesticide: |  |  |  |  |  |  |  |  |
| 9 | Harvesting: |  |  |  |  |  |  |  |  |
| 10 | Threshing & cleaning: |  |  |  |  |  |  |  |  |

**3.Indicator IV and V nutrient (N- & P- use efficiency)**

1. Basal and topdressing fertilizer application date, amount and nutrient content of the fertilizer

| SN | Fertilizer name | Days after seeding or transplanting | Applied amount (kg) | Nutrient content in the fertilizer | | |
| --- | --- | --- | --- | --- | --- | --- |
|  |  |  |  | N % | P_2_O_5_% | K_2_O% |
| 1. | Basal | | | | | |
| a. | Urea . . . . . . |  |  |  |  |  |
| b. | . . . . . . . |  |  |  |  |  |
| c. | . . . . . . . |  |  |  |  |  |
| d. | . . . . . . . |  |  |  |  |  |
| 2. | 1^st^ topdressing | | | | | |
| a. | . . . . . . . |  |  |  |  |  |
| b. | . . . . . . . |  |  |  |  |  |
| 3. | 2^nd^ topdressing | | | | | |
| a. | . . . . . . . |  |  |  |  |  |
| b. | . . . . . . . |  |  |  |  |  |
| 4. | 3^rd^ and all other later topdressings | | | | | |
| a. | . . . . . . . |  |  |  |  |  |
| b. | . . . . . . . |  |  |  |  |  |

1. If Zinc, sulfur or iron fertilizers are applied? If yes,

| SN | Element | Fertilizer name | Applied amount, (kg)  **Guess/best estimage the yield without any fertilizer:** |
| --- | --- | --- | --- |
| 1. | Zinc |  |  |
| 2. | Sulphur |  |  |
| 3 | Iron (Fe) |  |  |

**4. Indicator VI total water productivity**

Did you apply any irrigation during the rice growing period in the season? Yes…….. No………….

If yes, please specify in below events:

| SN | Irrigation events | Number | Depth (cm) |
| --- | --- | --- | --- |
| 1. | Irrigation applied to nursery: |  |  |
| 2. | Irrigation applied for puddling (land preparation + puddling): |  |  |
| 3 | Irrigation after transplanting to harvest: |  |  |

**5. Indicator VI Greenhouse gas emission**

1. What was the crop before kharif /wet season?

| Fallow | Rice with continuous flooding throughout season | Rice with intermittent flooding | Pulses | Vegetables | Others(name) |
| --- | --- | --- | --- | --- | --- |

b) Did you apply any organic amendment? Yes/No

c) Amount of organic amendments returned to the field (e.g., straw/residue, compost, FYM or green manure,

(for detail on types of organic amendments see below question) (kg)/acre.

|  |
| --- |

d) Type and time of organic amendments returned to the field.

| Pre-rice crop’s straw incorporated shortly (<30 days) before this rice cultivation | Pre-rice crop’s straw incorporated long (>30 days) before this rice cultivation | Compost | Farm yard manure | Green manure |
| --- | --- | --- | --- | --- |

e) Water regime during the cultivation in kharif season.

| Deep water | Flooded | Mostly flooded except 1-2 drying | Frequent dry spell | Drought |
| --- | --- | --- | --- | --- |

**6. Indicator VIII pesticide use efficiency**

a) Quantitative information

If yes, please fill up the information in the table.

| **Pesticide** | **Yes/No** | **No. of Applications** | **Product Name** | **Amount applied**  **(ml or gm)** | **a.i. % in the pesticide** |
| --- | --- | --- | --- | --- | --- |
| Herbicide | Yes/No |  | a. |  |  |
|  |  |  | b. |  |  |
|  |  |  | c. |  |  |
| Insecticide | Yes/No |  | a. |  |  |
|  |  |  | b. |  |  |
|  |  |  | c. |  |  |
| Fungicide | Yes/No |  | a. |  |  |
|  |  |  | b. |  |  |
|  |  |  | c. |  |  |
| Rodenticide | Yes/No |  |  |  |  |
| Molluscicide | Yes/No |  |  |  |  |

1. **Qualitative (scoreboard) information on pesticide use.**

| **Requirements** | **Level of performance (Please tick in one answer in each question)** |
| --- | --- |
| Use of registered products | 1. There is no use of pesticide 2. Purchased sealed product 3. Purchased loose product 4. Purchased for/from neighbor farmers 5. Purchase from input dealers |
|  |  |
| Targeted application | 1. Managed without pesticide 2. Aware of wind direction; hot or rainy days while planning for spray 3. Target crops for a pesticide |
|  |  |
| Label instructions | a) There is no use of pesticide  b) Farmers follow instructions mentioned on label for application method, and doses  c) Instructions followed on application method and pre-harvest intervals, but **suboptimal** doses  d) Incorrect application method, dosage in excess of labeled amount, or incorrect timing (how the farmer will say?) |
| Each pesticide application is in accordance with label instructions on application method, pre-harvest interval, and dosage. |  |
| Calibration | 1. There is no use of pesticides 2. Calibration and maintenance within current crop cycle 3. Calibration and maintenance within the past 2 years 4. No calibration and maintenance within the past 2 years 5. Outsource the service |
| Pesticide application equipment is calibrated, and it is maintained to prevent leakage or contamination of products. |  |
| Weed management | 1. Good land preparation 2. Flooding 3. Mechanical weeding 4. Manual weeding 5. Biological control agents |
| Which non-chemical method you practice? |  |
| Which Integrated weed management principle you follow? | 1. Herbicide is applied during the early crop growth stage before the rice canopy closes and when weeds are small. 2. An appropriate herbicide is used for the type of weed problem (choice of mode of action). 3. Local information about herbicide-resistant weeds is used when choosing an appropriate herbicide. |
| Insect management: was there insect infestation? | Yes/No |
| Which non-chemical method you practice? | 1. Synchronized planting 2. Use of resistant/tolerant varieties 3. Promotion of beneficial natural enemies (e.g., insects, spiders) by avoiding insecticide use 4. Promotion of other predators (e.g., birds, bats, frogs) 5. Crop rotation or extended fallow period 6. Balanced nutrient application (avoiding excessive use of nitrogen) 7. Biological control agents (e.g., *Metarizhium, Beauveria*) |
| Disease management: Any disease attack in the season?  If Yes, do you recognize the disease? | Yes/No  Yes/No, or If yes |
| Which non-chemical method you practice? | 1. Use resistant varieties 2. Synchronize planting 3. Remove host plants (weeds on bunds, rice stubble or volunteer rice) 4. Keep the environment between the soil and plant canopy either dry or moist (depending on the disease) 5. Plant at low densities 6. Use balanced nutrient application (avoiding excessive use of nitrogen) 7. Use biological control agents (e.g., Trichoderma) |
| Mollusk management: Was there any mollusks problem in your field? | Yes/No  If yes, |
| Which non-chemical method you practice? | 1. Physical control (vigilant destruction of egg masses, hand-picking of snails, baiting and capturing, maintaining saturation without standing water during the vulnerable period) 2. Promotion of predators (birds) 3. Use of sturdier seedlings during transplanting 4. Crop rotation or extended dry fallow period |
| Rodent management: Was there any rodent problem in your field? | Yes/No,  if yes, |
| Which non-chemical method do you practice? | 1. Synchronized planting 2. Community rodent management, for example, irradiation campaigns, trap crops 3. Using a trap crop 4. Trapping 5. Hunting 6. Use of narrow bunds (minimize rodent habitat) 7. Promotion of predators (birds of prey, snakes) |
| Bird management: Was there any birds’ problem in your field? | Yes/No  If yes, |
| Which non-lethal bird control option you practice? | 1. Synchronized planting 2. Scare/deterrent devices 3. Promotion of predators (e.g., birds of prey, shrikes) |

**7. Indicator IX: Energy use efficiency**

Machinery use

| **Operation** | **Source of power** | **Fuel** | **Time (hr) for 1 acre area** |
| --- | --- | --- | --- |
| Land leveling | 1. Animal 2. Two wheel tractor 3. 4 WT- 40 HP 4. 4 WT- 50 HP 5. 4 WT- 60 HP 6. 4 WT >60 HP | Diesel |  |
| Nursery bed preparation | 1. Animal 2. Two wheel tractor 3. 4 WT- 40 HP 4. 4 WT- 50 HP 5. 4 WT- 60 HP 6. 4 WT >60 HP | Diesel |  |
| Main field preparation | 1. Animal 2. Two wheel tractor 3. 4 WT- 40 HP 4. 4 WT- 50 HP 5. 4 WT- 60 HP 6. 4 WT >60 HP | Diesel |  |
| Sowing/Transplanting | 1. Manual 2. Two wheel tractor 3. 4 WT- 40 HP 4. 4 WT- 50 HP 5. 4 WT- 60 HP 6. 4 WT >60 HP | Diesel |  |
| Harvesting | 1. Manual 2. Reaper 3. Combine | Diesel |  |
| Threshing | 1. Manual 2. Animal 3. Paddle thresher 4. Axial flow thresher 5. Power thresher | Diesel  electricity |  |

**8. Indicator X Food safety**

| Are you aware of any risk in food safety (risks from arsenic, cadmium, chromium, mercury, and lead) | Yes | No |
| --- | --- | --- |
|  |  |  |
| If yes, please specify which heavy metal’s risk is there? |  | |
| If yes, whether they have done any soil remediation? |  | |

**9. Indicator XI Health and safety**

| **Requirements** | **Level of performance (Please tick in one answer in each question)** |
| --- | --- |
| Incidence of work related accidents | Yes/No, if yes, |
| The frequency of work-related accidents resulting in minor and major injuries or ill health for workers or any person on or outside the farm. | 1. No minor or major work-related injuries or ill health 2. No major work-related injuries or ill health, but minor cases in a lower frequency than in the last crop cycle 3. Any major work-related injuries or minor cases in an equal or higher frequency than in the last crop cycle |
| Do you have first aid box? | Yes/No, if yes, |
| Workers, including working household members, receive regular safety instruction to prevent work-related accidents or diseases, and first aid supplies are available on-farm. | 1. first aid supplies are available on-farm 2. first aid supplies are available at house 3. At both places (farm and house) 4. There is no first aid supplies are available on-farm |
| Calibration | 1. Calibration and maintenance within current crop cycle 2. Calibration and maintenance within the past 2 years 3. No Calibration and maintenance within the past 2 years 4. Outsource the service |
| Calibration of seed drill/machine transplantor |  |
| Training pesticide applicators | 1. There is no use of pesticides 2. Pesticide applicators participated in a training session in the past 1 years 3. Pesticide applicators participated in a training session in the past 3 years 4. Pesticide applicators participated in a training session in the past 5 years 5. Pesticide applicators did not participate in a training session in the past 5 year |
| Pesticide applicators receive training on handling and use of pesticides. |  |
| Personal protective equipment | 1. There is no use of pesticide 2. Pesticide applicators use **at least three** among (glove, mask, boots, protective clothing), but always use gloves of good quality, and clothing is washed after use 3. Pesticide applicator use **at least two** (gloves, masks, boots, protective clothing), but always gloves of good quality, and clothing is washed after use 4. Pesticide applicators use fewer than two of the four items, or do not use gloves, or use items of low quality, or clothing is not washed after use |
| Pesticide applicators use good-quality PPE, including gloves, masks, boots, protective clothing.  Protective clothing is washed after use. |  |
| Applicator restrictions | 1. There is no use of pesticide 2. Pesticides are not applied by pregnant or lactating women or by children below 18 years age, or by person who suffer from chronic or respiratory diseases 3. Pesticides are applied by pregnant or lactating women or children below 18 years, or by person who suffer from chronic or respiratory diseases |
| Pesticides are not applied by pregnant or lactating women, by children below 18 years, or by persons who suffer from chronic or respiratory diseases. |  |
| Re-entry time | 1. There is no use of pesticide 2. The recommendation, or re-entry after 48 hours is observed and communicated by placing warning signs in the field 3. The recommendation, or re-entry after 48 hours is observed and communicated verbally 4. The recommendation, or re-entry after 48 hours is not observed or not communicated |
| Recommended re-entry time after the use of pesticides, or after 48 hours if the label does not give a recommendation, is observed and communicated. |  |
| Pesticide storage | 1. There is no use of pesticide or inorganic fertilizers 2. Pesticides and inorganic fertilizers are labeled and stored in a **locked** and separate place 3. Pesticide and inorganic fertilizers are labeled and stored in a **general farm storage area** 4. Pesticides and inorganic fertilizers are **not** labeled or stored |
| Pesticides and inorganic fertilizers (including empty containers) are labeled and stored in a locked place, separate from fuel and food and out of the reach of children. |  |
| Pesticide disposal | 1. There is no use of pesticide 2. Farmer participate in a collection, return, or disposal system 3. Empty containers are rinsed three times with water and made unusable by crushing or puncturing before burying them on the farm and are not recycled 4. Empty container are rinsed and re used. 5. Surplus spray and wash water is applied over an unmanaged part of the farm, away from water bodies 6. Obsolete pesticides (date expired and banned) are returned to dealers or, if not possible, disposed of in a manner that minimize exposure to humans and environment 7. There is a collection, return, or disposal system, but it is not used. In the absence of such a system, empty pesticide containers and obsolete pesticides are not disposed as explained above |
| Empty pesticide containers and obsolete pesticides are properly disposed of. |  |

**10. Indicator XII Child labor**

| Do your kids (<15 years) get involve for this technology | Yes | No | |
| --- | --- | --- | --- |
|  |  |  | |
| If Yes, what type of work they do |  | | |
| Do labor brings their kids (<15 years) on work to take their help | Yes | | No |
| If Yes, what type of work they do |  | | |
| Children living on the farm (farmers kids or permanent labour kids) go to school | 1. There are no children living on the farm within the age of compulsory schooling 2. Children living on the farm within the age of compulsory schooling go to school all year long 3. Children living on the farm within the age of compulsory schooling, but not all year long 4. Children do not go to school but deliberate and evidenced efforts are made to place them in education, for example, by lobbying for a nearby school or by providing on-site schooling. | | |

**11. Indicator XIII Women empowerment (This will relevant to the selected technology)**

| **SN** | **Requirements** | **Level of performance (Please tick in one answer in each question)** |
| --- | --- | --- |
| 1. | Women's control over decisions regarding household agricultural production | 1. Women have at least equivalent decision-making power 2. Women have some but less than equivalent decision-making power 3. Women have no or marginal decision-making power |
|  | Women should have decision-making power over the choice of the products and markets of the household’s agricultural production. |  |
| 2. | Women's role and control over decision regarding selected technology | 1. Updated/familiar on adoption of new technology 2. Not updated/familiar on adoption of new technology |
| 3. | What has been changed for women with new technology | 1. Decision-making power increased 2. Decision-making power decreased 3. Their own labor input decreased 4. Their own labor input increased |
| 4. | Women's satisfaction regarding new technology | 1. Not involved 2. Good for increasing income 3. Good for Men but not for women 4. Good for both men & women 5. Hazardous |
| 5. | Women's satisfaction regarding their labor input | 1. Women are satisfied 2. Women are partly satisfied (e.g., no balance during peak labor-requirement periods) 3. Women are structurally unsatisfied |
|  | Women’s labor input in agricultural production should be in balance with their productive and domestic tasks, leisure, and possible other income-generating activities. |  |
| 6. | Women's access to information and capacity building for new technology | 1. Women have equal access 2. Women have less access 3. Women have no access |
|  | Women should have access to information, training, and extension services related to women’s activities. |  |
| 7. | Women's access to seasonal resources for farm activities | 1. Women have at least equivalent decision-making power and equal access 2. Women have some but less than equivalent decision-making power and less than equivalent access 3. Women have no or marginal decision-making power and no access |
|  | Women should have decision-making power and equal access to seasonal resources for farm activities, including hired labor, seeds, fertilizers, pest control products, and credit. |  |
| 8. | Women's control over decision-making regarding household income | 1. Women have at least equivalent decision-making power 2. Women have some but less than equivalent decision-making power 3. Women have no or marginal decision making power |
|  | Women and men should have decision-making power for the total household income. |  |
| 9. | Women's control over their personal income | 1. Women have equivalent or greater control 2. Women have some but less than equivalent control 3. Women have no or very limited control |
|  | Women should have equivalent or greater control of income they generate themselves. |  |
| 10. | Women's participation in collective decision-making | 1. Women participation in group leadership, are active in group decision, and their voices are valued 2. Women are present during group decision, but their contributions are not given full weight 3. Women are excluded from group decision-making |
|  | Women should be able to participate in group decision making processes regarding rice production and marketing (e.g., irrigation scheduling, leadership committees of cooperatives, credit groups). |  |
| 11. | Violence against women | 1. There are no cases of violence 2. There is at least one case of violence |
|  | There must not be any violence that results in, or is likely to result in, physical, sexual, or mental harm or suffering to women, including threats of such acts, coercion, or arbitrary deprivation of liberty, whether occurring in public or in private life (UN resolution 48/104 of 20 December 1993). |  |

**Appendix 2.**

1. **Computation of 12 SRP performance indicators (PIs)**

All 12 performance indicators, i.e. net profit, labor productivity, grain yield, food safety, water productivity, nitrogen-use efficiency (NUE), phosphorus-use efficiency (PUE), pesticide-use efficiency, GHG emission, health and safety, child labor, and women empowerment were calculated as defined by SRP (SRP 2015a).

- ***Net profit ($ ha^-1^):*** Net profit (profitability) for each household was computed by subtracting all variable costs of inputs (seed, fertilizer, pesticide, irrigation), labor (family and hired), and machinery (hired or own) incurred during nursery and field preparation to crop harvesting, threshing, cleaning and drying, and land rent (if any) from the total income from fresh grain and straw yield.
- ***Labor productivity (kg paddy labor d^-1^):*** To compute labor productivity, both hired and own (family) labor of both male and female were considered, and labor productivity was estimated by dividing rice yield by the number of labor used to produce in a season.
- ***Grain yield (kg ha^-1^):*** Grain yield was computed based on well-sun dry basis as farmers in the region do not have access of measuring grain moisture content.
- ***Food safety:*** The indicator of food safety requires laboratory analysis of heavy metals, e.g., arsenic, cadmium, mercury, chromium, and lead, and pesticide residues. As we did not have access to a high-quality service laboratory for these analyses, this indicator was computed based on the farmer's response to the reported problem in the rice field (SRP, 2015a).
- ***Water productivity:*** Though the SRP indicators used the term “water use efficiency” but they mean it as “water productivity”. To keep in consistent, we have used the term water productivity in this paper. Water productivity (kg paddy L-1 irrigation + rainfall) was computed by diviging the grain yield by dividing total amount of irrigation and rainfall water (L). The total amount of irrigation water applied was computed from the number of irrigation and the approximate depth of irrigation applied from the start of field preparation to crop harvest, and the total amount of rainfall computedfrom crop sowing/transplanting to harvest (crop growing period) for each farmer using the rainfall data obtained from the Odisha Meteorological stations.
- ***Nitrogen and phosphorus-use efficiency (kg paddy kg^-1^ elemental N or P)****:* Nitrogen and phosphorus-use efficiencies were computed based on the elemental form of the nutrient elements. To compute P use efficiency, P_2_O_5_ fertilizers were multiplied by a factor of 0.4364. Then, the total grain yield harvested was divided by the elemental N or P and the efficiency expressed in terms of kg grain kg^-1^ elemental N or P.
- ***Pesticide-use efficiency:*** The pesticide use efficiency was calculated using the SRP scorecard, which is based on the number and the timing of application of different categories of pesticide, i.e., herbicide, insecticide, fungicide, rodenticide, molluscicide, and avicides (SRP, 2015a; Stuart et al., 2018). Bird control was also included in the computation of the pesticide use score. For the assessment, the scorecard values were categorized (out of a possible score of 100) as:, “Gold” if the scorecard value ≥ 80, “Acceptable” if 65-80, “Tolerable” if 50-65, and “Unsustainable” if ≤ 50.
- ***Greenhouse gas emission:*** Greenhouse gas emission was computed using the formulas given by IPCC (Dong et al., 2006) and Stuart et al. (2018). As described in Dong et al. (2006), all the information required for the computation of GHG, i.e., water regime (flooding period) before rice planting, amount and type of organic material returned to the field and the organic material incorporation time, irrigation method and water regime during rice growing period, growing duration of the variety, and the default emission factor of 1.3 were used.
- ***Worker health and safety, child labor, and women empowerment:*** These three indicators were computed using scorecard (SRP, 2015a). For all these indicators, the scorecard values were ranked (out of a possible score of 100) as, “Good” if the scorecard value ≥ 80, “Fair” if 50-80, “Poor” if ≤ 50.

1. **Computation of energy use efficiency**

Besides the computation of the 12 SRP indicators, we also computed and compared the energy use efficiency (a potential 13^th^ indicator for consideration by SRP) among three rice establishment methods. For all three establishment methods, agronomic energy input (AEI), i.e., all energy inputs for production, machinery, diesel, human labor, animal power, and inputs (seed, fertilizers, pesticides); energy output/grain yield energy (GYE), i.e., yield values in-terms of energy; net energy balance (NEY), i.e., the difference between output and input; and net energy yield productivity (NEYP), i.e., GJ energy t^-1^ rice, for different methods of rice establishments were computed. Questions on the type of machines used, technical specification of machineries including motor capacity (horsepower), type of fossil fuel consumption during operation, and the total time (h) taken for the particular machinery for particular operations, e.g., laser land leveling, nursery bed preparation, main field preparation, and direct seeding or transplanting, harvesting (manual or reaper or combine), and threshing in the surveyed rice area were asked. When computing AEI, energy consumed by machines during these field operations were computed using the modifying formula (below) provided by Eskandari and Attar (2015) as these services were mostly provided by the service providers and thus the efficient life-time of the machine and the power-to-weight ratio (some machines are old) are beyond the control and can vary from one service provider to another.

$$Energy consumed by machine \left( {MJ ha}^{-1} \right)= Machine horse power \left( hp \right) X 44.8 X 0.223 X Time of machinery uses \left( {h ha}^{-1} \right)\ldots\ldots\ldots\ldots. (1)$$

Where, the value 44.8 is the energy conversion equivalent for diesel (SI Table 2) and 0.223 is the L diesel consumption hp-h (Eskandari and Attar, 2015).

Also for the same land area, planting and harvesting method (manual or machinery), crop yield per unit area, number and the approximate depth of irrigation (volume of irrigation water in m^3^ ha^-1^ during the crop growing period computed, Fig. 6), number and type (male or female) of manual labor (person-hour) starting from nursery and land preparation to harvesting and threshing (Fig. 4), the amount of seed, amount and type (nitrogen, phosphorus, potassium, Fig. 5) of fertilizers, and amount and type of pesticides (herbicide, insecticide, fungicide, rodenticide, molluscicide) used, and amount of compost applied wererecorded through recall during survey. Using these information, four below indicators related to energy were computed as:

$$Grain yield energy \left( GYE;{GJ ha}^{-1} \right)=Harvested rice yield \left( {t ha}^{-1} \right) X Rice grain energy conversion \left( SI Table 2 \right)\ldots\ldots\ldots\ldots\ldots.(2)$$

$$Agronomic energy input \left( {AEI; GJ ha}^{-1} \right)=Energy from all input variables \left( Inputs x energy conversion factor;SI Table 2 \right)+ energy consumed by the machines in different field operations \left( SI Table 3 \right)\ldots\ldots\ldots\ldots\ldots..(3)$$

$$Net energy yield \left( NEY; {GJ ha}^{-1} \right)=Energy output \left( GYE \right)-Energy input \left( AEI \right)\ldots..(4)$$

$$Net energy yield productivity \left( NEYp; {GJ ha}^{-1} \right)=\frac{Net energy yield (NEY)}{Grain yield ({t ha}^{-1})}\ldots\ldots\ldots\ldots.(5)$$
